# The effectiveness of the quality improvement collaborative strategy in low- and middle-income countries: A systematic review and meta-analysis

**DOI:** 10.1371/journal.pone.0221919

**Published:** 2019-10-03

**Authors:** Ezequiel Garcia-Elorrio, Samantha Y. Rowe, Maria E. Teijeiro, Agustín Ciapponi, Alexander K. Rowe

**Affiliations:** 1 Healthcare quality and safety department, Instituto de Efectividad Clínica y Sanitaria (IECS-CONICET), Buenos Aires, Argentina; 2 Malaria Branch, Division of Parasitic Diseases and Malaria, Center for Global Health, Centers for Disease Control and Prevention, Atlanta, Georgia, United States of America; 3 CDC Foundation, Atlanta, Georgia, United States of America; 4 Quality Department, Fundación para la Lucha contra las Enfermedades Neurológicas de la Infancia (FLENI), Escobar, Buenos Aires Province, Argentina; 5 Argentine Cochrane Centre, Instituto de Efectividad Clínica y Sanitaria (IECS-CONICET), Buenos Aires, Argentina; University of Mississippi Medical Center, UNITED STATES

## Abstract

**Background:**

Quality improvement collaboratives (QICs) have been used to improve health care for decades. Evidence on QIC effectiveness has been reported, but systematic reviews to date have little information from low- and middle-income countries (LMICs).

**Objective:**

To assess the effectiveness of QICs in LMICs.

**Methods:**

We conducted a systematic review following Cochrane methods, the Grading of Recommendations Assessment, Development, and Evaluation (GRADE) approach for quality of evidence grading, and the Preferred Reporting Items for Systematic Reviews and Meta-analyses (PRISMA) statement for reporting. We searched published and unpublished studies between 1969 and March 2019 from LMICs. We included papers that compared usual practice with QICs alone or combined with other interventions. Pairs of reviewers independently selected and assessed the risk of bias and extracted data of included studies. To estimate strategy effectiveness from a single study comparison, we used the median effect size (MES) in the comparison for outcomes in the same outcome group. The primary analysis evaluated each strategy group with a weighted median and interquartile range (IQR) of MES values. In secondary analyses, standard random-effects meta-analysis was used to estimate the weighted mean MES and 95% confidence interval (CI) of the mean MES of each strategy group. This review is registered with PROSPERO (International Prospective Register of Systematic Reviews): CRD42017078108.

**Results:**

Twenty-nine studies were included; most (21/29, 72.4%) were interrupted time series studies. Evidence quality was generally low to very low. Among studies involving health facility-based health care providers (HCPs), for “QIC only”, effectiveness varied widely across outcome groups and tended to have little effect for patient health outcomes (median MES less than 2 percentage points for percentage and continuous outcomes). For “QIC plus training”, effectiveness might be very high for patient health outcomes (for continuous outcomes, median MES 111.6 percentage points, range: 96.0 to 127.1) and HCP practice outcomes (median MES 52.4 to 63.4 percentage points for continuous and percentage outcomes, respectively). The only study of lay HCPs, which used “QIC plus training”, showed no effect on patient care-seeking behaviors (MES -0.9 percentage points), moderate effects on non-care-seeking patient behaviors (MES 18.7 percentage points), and very large effects on HCP practice outcomes (MES 50.4 percentage points).

**Conclusions:**

The effectiveness of QICs varied considerably in LMICs. QICs combined with other invention components, such as training, tended to be more effective than QICs alone. The low evidence quality and large effect sizes for QIC plus training justify additional high-quality studies assessing this approach in LMICs.

## Introduction

Major failures in health care have been reported elsewhere but are most evident in low- and middle-income countries (LMICs). An evaluation of the health-related Millennium Development Goals (MDGs) found that, in 2015, when they were to be achieved, major health care quality gaps still were present in LMICs, which ignited a strong demand for quality improvement [1]. The MDGs have now been replaced by the Sustainable Development Goals (SDGs), instituted by the United Nations with the aim to contribute to the achievement of universal health coverage with quality care for all [2]. Concurrently in 2017, The Lancet Global Health Commission on High-Quality Health Systems in the SDG Era was established to review current knowledge, conduct new focused research, and propose policies for measuring and improving health care quality to reach new levels of performance in LMICs. This Commission advocated for a revision of methods that could contribute to the advance of the field of quality of care worldwide [3].

Among the several quality improvement strategies available, quality improvement collaboratives (QICs) (also known as collaborative improvement and learning collaboratives) have been used to improve health care for several decades [4]. However, reporting on specific components of QICs has been imprecise [5].

Formal QICs involve the use of healthcare teams from different sites to improve performance on a specific topic by collecting data and testing ideas with improvement cycles (usually plan-do-study-act cycles, involving planning a change, trying it, observing the results, and acting upon what is learned) supported by coaching and learning sessions [6]. QICs are supported by the concept that district managers and networks of facilities can be harnessed into learning systems that accelerate improvement in health care performance with the potential to achieve results at large scale for scale. The district level of the health system is well positioned to facilitate systematic group learning among facilities of similar types and across tiers of the health system. District-led area-based learning and planning bring together providers and administrators responsible for a catchment area to solve clinical and system problems, harmonize approaches, maximize often limited resources and create better communication and referral between facilities [7].

The use of QICs has increased rapidly despite the absence of strong evidence for effectiveness, cost-effectiveness or long-term impact. Published systematic reviews on QICs, which predominantly include studies from high-income countries, show modest improvements, particularly when addressing straightforward aspects of care where there is a clear gap between recommended and actual practice. There is still limited information from LMICs, unpublished studies, or non-English studies [8–10].

Recently, an extensive systematic review has been published characterizing the effectiveness of a wide array of strategies to improve health care provider (HCP) performance in LMICs (the Health Care Provider Performance Review, or HCPPR) [11]. Although this review includes QICs, thus far, these strategies have been analyzed under the broader strategy category of “group problem solving,” which includes other, non-QIC, strategies. Additionally, the most recent literature search for the HCPPR was conducted in May 2016.

The objective of this work was to particularly estimate the effectiveness of QICs in LMICs using data from the HCPPR and results of studies from an updated literature search. We aimed to inform decisions about whether to use QIC, how best to implement them, and to identify knowledge gaps on QICs in LMICs and provide direction on future evaluations of this strategy.

## Materials and methods

We conducted a systematic review following Cochrane Collaboration methods and the Preferred Reporting Items for Systematic Reviews and Meta-analyses (PRISMA) statement for reporting [12, 13]. The study protocol was registered in PROSPERO International prospective register of systematic reviews (registration number CRD42017078108).

### Study eligibility criteria

#### Type of study designs

Studies meeting the Cochrane Effective Practice and Organisation of Care (EPOC) Review Group for inclusion in a systematic review of interventions [14]:

- Randomized controlled trials (RCTs)
- Controlled before- and- after trials (CBA)
- Interrupted time series (ITS) designs with at least 3 data points before and after the intervention, with or without comparison groups

#### Types of participants

HCPs (and patients that they care for) from LMICs (defined as countries with a low or middle-income economy, according to the World Bank at the time of the literature search) [15]. HCPs included hospital-, clinic-, and community-based health workers, pharmacists, and medicine vendors.

#### Type of intervention

Studies were included if they had an intervention arm exposed to QIC with or without other strategy components (e.g., training) compared to a non-exposed control group (or historical controls, for ITS studies) that could be defined as usual practice. QIC was defined as a strategy with the following core elements: a) a team of experts (in clinical care and quality improvement) involved in bringing together the scientific evidence, practical contextual knowledge and quality improvement methods, usually within a “change package” or toolkit; b) multiple teams from multiples sites that chose to participate; c) a model or framework for improvement that included measurable aims, data collection, implementation and evaluation of small tests of change; and d) a set of structured activities that promoted a collaborative process to learn and share ideas, innovations, and experiences (e.g. face-to-face or virtual meetings; visits to other sites; visits by experts or facilitators; web-based activities to report changes, results and comparisons with other teams; and coaching and feedback by improvement experts). The comparator was non-exposed control groups that represent usual practice.

#### Type of outcomes

There was no restriction on outcome type. Outcomes were grouped into the following categories.

- Facilitators (i.e., elements that facilitate HCP performance, such as supplies and HCP knowledge)
- Health worker practices (i.e., processes of care, such as correct treatment)
- Patient health outcomes
- Patient behaviors related to care-seeking or use of health services
- Other patient behaviors (i.e., those not related to care-seeking, such as adherence to treatment regimen)

Effect sizes were based on primary outcomes, with the following exclusions.

- For outcomes expressed as a percentage, effect sizes based on <20 observations per study group and time point, for a given comparison
- Effect sizes based on a simulation study and not actually observed data
- Effect sizes for which baseline and follow-up measures in the intervention group were both 100%, as this indicates that HCP performance in the intervention group had no room for improvement and did not worsen over time. Similarly, for HCP practice outcomes expressed as a percentage, we excluded effect sizes based on a baseline value of 95% or greater, as there was little room for improvement.
- Effect sizes based on outcome measures that were not taken at comparable times between study groups. For example, if the outcome for a control group was measured at –1 month, 3 months, and 9 months since the intervention began, and the outcome for an intervention group was measured at –1 month, 3 months, and 21 months since the intervention began, the effect size based on the 9-month and 21-month outcome measures would be ineligible.
- Outcomes from ITS studies for which the time series was highly unstable and thus could not be reliably modeled, and outlier outcome measures that probably did not represent the true trend in HCP performance.

### Search strategy

**Fig 1:**
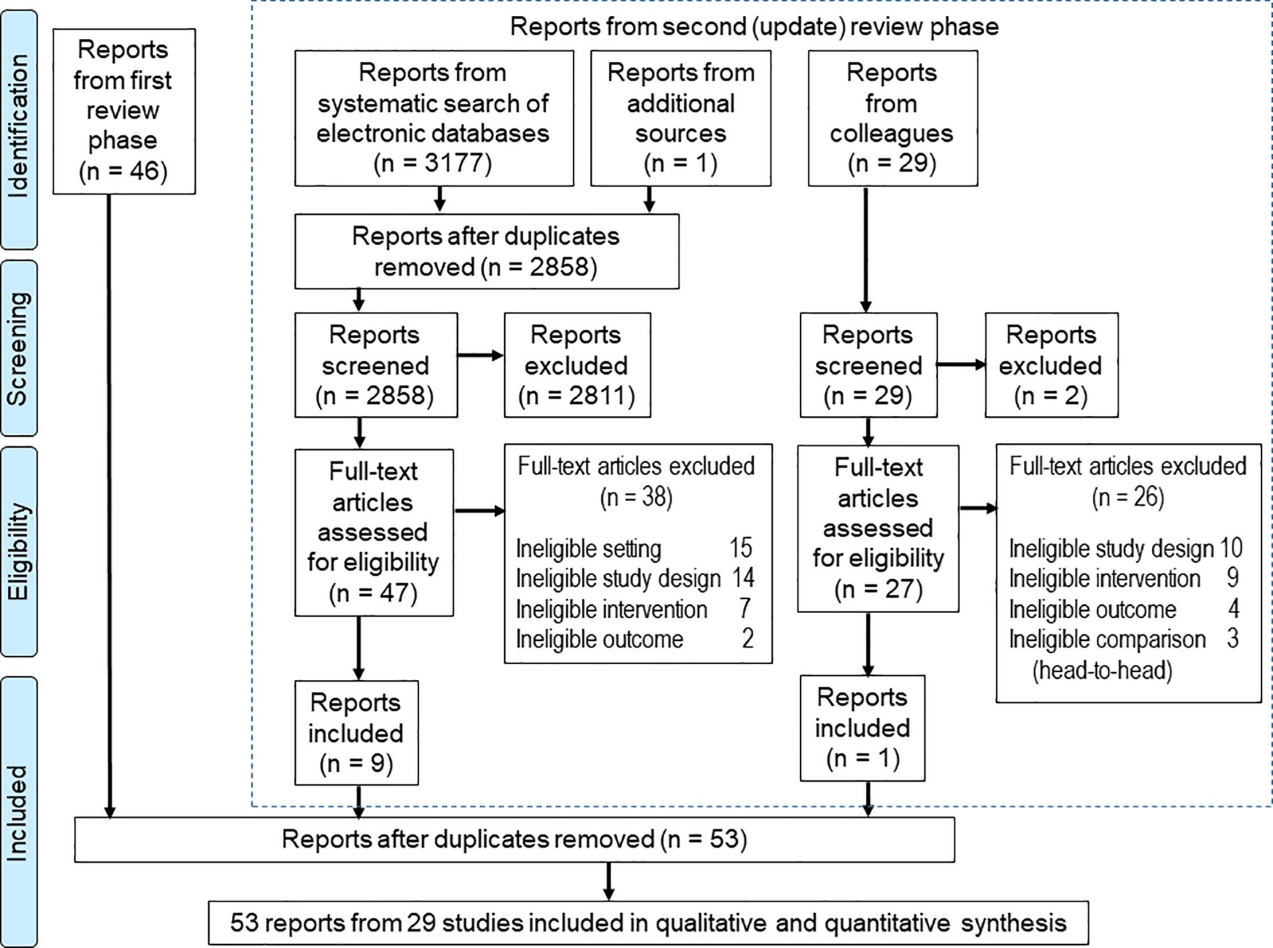
Flow diagram.

The literature search was conducted in two phases (see S1 File for details). In summary, we first searched results of the HCPPR, which is a comprehensive systematic review of the effectiveness of strategies to improve health worker performance in LMICs. The HCPPR study team searched 52 electronic databases for published studies and 58 document inventories for unpublished studies from 1960s–2016, screened personal libraries, asked colleagues for unpublished studies, and performed hand searches of 854 bibliographies from previous reviews. Second, we updated the HCPPR literature search with a focus on studies of QICs (search date was March 15, 2019). This update involved the search of electronic databases (S1 File, page 14), screening bibliographies of included study reports (referred to as “reports from additional sources” in Fig 1), and seeking reports from colleagues. There were no language restrictions.

### Data collection

In the first phase of the review, a team of researchers assessed study eligibility, and each researcher screened studies independently. Before the screening began, concordance testing was conducted against a “gold standard” list of reports until at least 80% was identified by each researcher. In the second phase of the review, a pair of investigators (MET, EGE) independently assessed study eligibility, and discrepancies were reconciled in consultation with a third team member (AC). The study eligibility process was conducted using Covidence© from the Cochrane collaboration. Also, two investigators (AKR, SYR) assessed the eligibility of study reports that we received from colleagues. Data were extracted from the included studies independently by a pair of investigators (SYR, AKR) or researchers using a standardized form, and discrepancies were resolved through discussion. Before beginning data extraction, concordance testing of all data abstractors was conducted until the percent agreement between individual abstractors and a gold standard set of abstracted data (based on consensus by investigators SYR, AKR) was at least 80%. Data from each study were entered into a Microsoft Access database (Microsoft Inc., Redmond, Washington). Data elements included: study setting (where, when, HCP types, other contextual factors), study design, health conditions addressed, strategy description, outcome description, outcome measurements, the timing of outcome measurements in relation to the implementation of the strategy, effect sizes, sample sizes, sampling details, and data elements needed to assess risk of bias (RoB). If details regarding study characteristics or the QIC intervention were not available in study reports, we contacted study authors. Except for the purpose of meta-analysis, missing data were not imputed. For meta-analysis, we used estimates of standard errors of effect sizes that were available from the HCPPR database. A small proportion of the standard error estimates for percentage outcomes from the HCPPR database were based on imputed data (usually because sample size data were missing). Effect sizes with missing standard errors were excluded from meta-analysis.

### Risk of bias (quality) assessment

We categorized RoB with methods based on guidance from the Cochrane EPOC Group [16]. RoB at the study level was categorized as low, moderate, high, or very high. We assessed the following RoB domains: number of clusters per study arm, completeness of dataset, balance in baseline outcome measurements, balance in baseline characteristics, reliability of outcomes, adequacy of concealment of allocation (where relevant), intervention unlikely to affect data collection, intervention plausibly independent of other changes, and number of data points before and after the intervention.

We used the Recommendations Assessment, Development, and Evaluation (GRADE) approach to assess the quality of evidence related to each of the key outcomes [17]. For assessments of the overall quality of evidence for each outcome, randomized studies, ITS studies, and other non-randomized studies started at “high quality”, “moderate quality” and “low quality” of evidence, respectively. Although the traditional approach is to start non-randomized studies as “low quality” [18], ITS studies with multiple periods and measurements during each period with no other limitations may constitute “moderate quality” of evidence [19, 20]. We downgraded the study one or two levels depending on the extent of violation across the following criteria: study limitations (RoB); indirectness of evidence; inconsistency; imprecision of effect estimates; or publication bias. If we did not find study limitations, we upgraded the evaluation of the quality of the evidence when the pooled estimates revealed negligible concerns about confounders, a strong dose-response gradient, or a large magnitude of effect. Considering a mean baseline health worker performance level at 40% for a process-of-care outcome expressed as a percentage, an absolute increase of 40% or more, representing a relative risk >2, allowed us to upgrade the quality of evidence by one level.

### Data synthesis

Effect sizes were defined as absolute percentage-point differences; positive values meant improvement.

In non-ITS studies with pre- and post-intervention outcome measures, for outcomes that were dichotomous or expressed as a percentage, the effect size was calculated with Eq 1.

$$effect\;size={\left(followup-baseline\right)}_{intervention}-{\left(followup-baseline\right)}_{control}$$ (1)

In non-ITS studies with pre- and post-intervention outcome measures, for outcomes that were continuous but not obviously bounded (e.g., a mortality rate), the effect size was calculated with Eq 2.

$$effect\;size=100\%\left[{\left(\frac{followup-baseline}{baseline}\right)}_{intervention}-{\left(\frac{followup-baseline}{baseline}\right)}_{control}\right]$$ (2)

For ITS studies, segmented linear regression modeling was performed to estimate a summary effect size that incorporated both the level and trend effects. The summary effect size was the outcome level at the mid-point of the follow-up period as predicted by the regression model minus a predicted counterfactual value that equals the outcome level based on the pre-intervention trend extended to the mid-point of the follow-up period. This summary effect size was used because it allows the results of ITS studies to be combined with those of non-ITS studies.

To estimate strategy effectiveness from a single study comparison, the effect size was defined as the median of all effect sizes (MES) in the comparison for outcomes in the same outcome category. Results were stratified by HCP type (health facility-based vs. lay or community HCP).

For the primary analysis, we reported median, interquartile range, minimum, and maximum MES. The median effect size has been used in other systematic reviews of strategies to improve HCP performance [21, 22]. Median MES for strategy groups that were based on fewer than five study comparisons were not weighted, as weighting with small samples might cause the median to be a poor measure of central tendency when outliers are present. Median MES for strategy groups with five or more study comparisons were weighted, where the weight = 1 + the natural logarithm of the number of HCPs or (if the number of HCPs in a study was not reported) the number of service provision sites (e.g., health facilities) or (if the number of service provision sites was not reported) the number of administrative areas (e.g., districts) in the study. Strategy groups tested by at least three study comparisons were considered to have enough evidence to form generalizations—although caution is increasingly warranted as the minimum of three comparisons is approached. Strategy groups tested by only one or two study comparisons were interpreted separately.

In a secondary analysis, standard random-effects meta-analysis was used to estimate the weighted mean MES and 95% confidence interval (CI) of the mean MES of each strategy group. We used I^2^ as a measure of consistency for each meta-analysis, considering low heterogeneity <30%, moderate heterogeneity 30–60%, and high heterogeneity >60% [23]. We conducted a meta-analysis on one median effect size per study comparison for each outcome group, and we performed a sensitivity analysis considering all effect sizes individually to test consistency of the results.

Publication bias was assessed using Funnel Bias Assessment plots to conduct visual inspection for asymmetry for strategy-outcome groups with at least 10 studies.

## Results

During the first phase of the literature search, 216,477 citations were identified (S1 File). After screening and assessing eligibility, 46 reports from 25 studies were included (left side of Fig 1). In the second phase, which updated the search through 15 March 2019, 3207 articles were identified, and seven more reports from four studies were included after removing duplicates. Altogether, 53 reports from 29 studies with 30 study comparisons were included for this systematic review (Fig 1).

### Description of included studies

The included studies were published between 2008 and 2019, from 12 LMICs in four continents. Most studies (24/29, 82.7%) were from Africa, three were from the Russian Federation, and one each was from Georgia and Mexico (Table 1). Most studies were ITS studies without controls (19/29, 72.4%), two were CBAs with randomized controls, three were CBAs with non-randomized controls, two were post-only CRTs, and one was an ITS study with controls.

**Table 1:** Characteristics of included studies.

Study ID	Country	Study design	Intervention	Outcomes	Effect size ± standard error
**N’Guessan 2011 [27,28]**	Cote d'Ivoire	ITS	QIC	% of patients lost to follow-up during antiretroviral treatment	3.4±8.6
				% of HIV-exposed infants who were tested for HIV	15.5±20.7
				% of patients’ files with complete documentation for antiretroviral treatment	44.8±10.2
				% of patients’ files with complete documentation for prevention of mother to child transmission of HIV	76.7±3.8
**Chitashvili 2017 [29–34]**	Georgia	CBA (NRC)	QIC	% of hospital pediatric patients with antibiotic prescription who received appropriate first-line antibiotic for pneumonia	31.0±26.3
				% of clinic pediatric patients with antibiotic prescription for whom antibiotic was justified for respiratory tract infection	64.0±9.2
				% of clinic pediatric patients with antibiotic prescription who received appropriate first-line antibiotic for respiratory tract infection	68.0±8.7
**Singh 2013 [35–38]**	Ghana	ITS	QIC	Mean % of deliveries attended by a skilled birth attendant defined as a doctor, nurse or midwife per HF	-3.6±26.1
				Mean % of newborns who received follow-up post-natal care on day 6 or 7 after birth per HF	28.9±27.1
				Mean % of newborns who received post-natal care within 48 hours of birth per HF	29.0±30.6
				Mean % of infants attending child wellness clinics who were low weight for age per HF	116.1±71.3
**Singh 2016–1 [36–38–41]**	Ghana	ITS	QIC	Mean % of antenatal care registrants in the first trimester at the time of registration per HF	-0.997±3.8
				Mean % of total deliveries that are attended by skilled personnel per HF catchment area	10.5±4.8
				Mean % of 1- to 11-month-old child welfare clinics attendees who were < 60% weight for age (moderately or severely underweight) per HF	20.4±45.6
**Singh 2016–2 [36–38–41]**	Ghana	ITS	QIC	Mean % of antenatal care registrants in the first trimester at the time of registration per HF	-5.4±4.0
				Mean % of total deliveries that are attended by skilled personnel per HF catchment area	16.4±5.5
				Mean % of 1- to 11-month-old child welfare clinics attendees who were < 60% weight for age (moderately or severely underweight) per HF	71.7±60.8
**Singh 2016–3 [36–38–41]**	Ghana	ITS	QIC	Mean % of antenatal care registrants in the first trimester at the time of registration per HF	7.0±8.1
				Mean % of total deliveries that are attended by skilled personnel per HF catchment area	12.7±9.4
**Colbourn 2013 [42–44]**^a^	Malawi	CBA (RC)	QIC	Maternal mortality rate per 100000 livebirths	-27.4^b^
				Perinatal mortality rate per 1000 births (stillbirths and early neonatal deaths)	3.0^b^
				Neonatal mortality rate per 1000 livebirths	22.9^b^
**Barceló 2010 [45]**	Mexico	POS-CRT	QIC	% of patients who received eye examination	68.5±14.2
				% of patients who received foot examination	73.8±13.5
				% of patients with blood pressure < = 140/90 mmHg	5.8±18.4
				% of patients with A1c <7% (good diabetes control)	11.4±19.2
				% of patients with cholesterol <200mg/dL	17.9±18.9
				Average triglycerides per patient (mg/dL)	0.8^b^
				Average body mass index per patient (kg/m2)	2.0^b^
**Crigler 2012 [46–47]**	Niger	ITS	QIC	Number of deliveries assisted by skilled health workers per 100 expected pregnancies	-25.8±81.0
				Contraceptive prevalence rate (number of women who accepted contraceptive use at HF per 100 women of reproductive age in catchment area)	131.3±231.0
				% of HCPs with an adequate job description	47.2±139.0
				% of HCPs adhering to norms for essential newborn care at birth	8.1±24.1
**Osibo 2017 [48–50]**	Nigeria	POS-CRT	QIC	% of HIV positive pregnant women who attended 6-month postpartum visit and did not miss any previous scheduled visit by more than 30 days	3.0±12.8
**Catsambas 2008–4 [51–57]**	Russian Federation	ITS	QIC	% of deliveries to women with no pregnancy induced hypertension out of all deliveries per month	34.3±9.9
**Catsambas 2008–5 [51–57]**	Russian Federation	ITS	QIC	% of women who were pregnant this month with pregnancy induced hypertension of any severity for whom pregnancy induced hypertension protocol was implemented	49.7±26.4
				Outcome on diagnostic accuracy: ratio of number of deliveries to women with no pregnancy induced hypertension to number of all deliveries per month (x100%)	3.4±6.1
				Outcome on diagnostic accuracy: % of deliveries to women with no edema out of all deliveries per month	5.2±2.2
				Decrease = improvement: ratio of number of women hospitalized this month for pregnancy induced hypertension complications to number of women who completed pregnancy this month with pregnancy induced hypertension of any severity (x100%)	-75.3±63.9
**Catsambas 2008–6 [52,53,55,57]**	Russian Federation	ITS	QIC	% of patients with hypertension who were taken under observation in the first stage of disease	10.3±7.0
				% of patients with hypertension who were taken under observation who performed non-drug treatment recommendations	12.5±1.1
				number of patients with hypertension identified for the first time per 1000 residents of HF catchment areas	-28.2±45.0
				% of patients with hypertension who were taken under observation who have consistently reduced blood pressure	-15.0±9.8
**Catsambas 2008–7 [52,54,55,57,58]**	Rwanda	ITS	QIC	% of women who enrolled for antenatal care consultations and were tested for HIV whose male partners were also tested for HIV	-1.5±35.0
				% of women who enrolled for antenatal care consultations and were tested for HIV who returned for their results the same day of testing	44.6±29.0
**Ngidi 2013 [59]**	South Africa	CITS	QIC	mean number of antenatal clients referred for antiretroviral therapy per HF per month	-4.7^b^
				mean number of antenatal clients initiated on antiretroviral therapy per HF per month	172.9 ^b^
**Catsambas 2008–9 [52,54,55,57]**	Tanzania	ITS	QIC	% of women who tested positive for HIV who attended antenatal care consultations and were enrolled in Care and Treatment Center per month	-10.1±11.7
				% of HIV patients on antiretroviral therapy seen at clinic according to their scheduled appointments who were not lost to follow-up for at least 3 consecutive months	9.2±1.7
				% of HIV patients in general care or on antiretroviral therapy seen at clinic within past month who were assessed for active tuberculosis at every visit within past month	-3.8±1.8
				% of estimated number of HIV-exposed infants born in this month who received antiretroviral prophylaxis per month	21.3±34.2
				% of HIV patients on antiretroviral therapy who were seen in clinic within past month who had documented contact tracing information for 2 cohorts	27.2±16.6
				% of HIV patients in general care seen at clinic within past 6 months who had CD4 test once during those 6 months	34.6±21.2
				% of estimated number of HIV-exposed infants born in preceding 12 months who started receiving cotrimoxazole within 2 months of age	59.2±44.6
**Catsambas 2008–10 [52,54,55,57,58]**	Uganda	ITS	QIC	% of HIV patients on antiretroviral therapy seen at clinic within past month who were adherent to 95% or more of prescribed doses of antiretroviral medicines	26.2±7.5
				% of HIV patients in general care or on antiretroviral therapy seen at clinic within past month who were assessed for active tuberculosis at every visit within past month	19.6±13.8
**Catsambas 2008–11 [52,54,55,57,60]**	Uganda	ITS	QIC	% of HIV patients on antiretroviral therapy seen at clinic within past month who were adherent to 95% or more of prescribed doses of antiretroviral medicines	17.6±3.6
				% of HIV patients on antiretroviral therapy for past 6 months seen at clinic who showed clinical improvement (weight steady or increasing, ambulatory or better, no opportunistic illnesses)	0.3±8.5
**Catsambas 2008–12 [52,54,55,57,60]**	Uganda	ITS	QIC	% of HIV patients in general care or on antiretroviral therapy seen at clinic within past month who were assessed for active tuberculosis at every visit within past month	14.3±12.5
**Jaribu 2018 pilot [61]**	Tanzania	ITS	QIC	Median number of facility deliveries per facility per month	85.7±55.4
**Jaribu 2018 implementation [61–62]**	Tanzania	ITS	QIC	Median number of deliveries in which partographs with 4 assessment indicators completed per facility per month	135.2±120.4
				Median number of facility deliveries per facility per month	7.1±13.4
**Catsambas 2008–1 [52,55,57,63,64]**	Niger	ITS	QIC + training + poster for HCP	% of live births delivered vaginally in the maternity for which immediate breastfeeding within one hour after birth occurred	78.7±3.9
				% of acute management of third stage of labor standards met among total number of acute management of third stage of labor standards expected on the partographs analyzed	46.3±3.9
				% of newborns whose temperature was measured	60.9±7.7
				% of standards observed in essential newborn care among total criteria expected in cases analyzed	71.0±7.3
				% of vaginal deliveries performed in the maternity where the three elements of active management of third stage of labor were applied	91.4±2.6
				Decrease = improvement: number of stillbirths per 1000 births in maternity (vaginal and cesarean)	16.9±22.4
				Decrease = improvement: Number of neonatal deaths by time of discharge from hospital per 1000 children born at home or in the maternity (vaginal and cesarean)	39.7±52.9
				Decrease = improvement: number of women who suffered from postpartum hemorrhages per 1000 women who delivered vaginally in the maternity	96.0±20.1
				Decrease = improvement: Number of all-cause maternal deaths per 1000 births (vaginal or cesarean) in the maternity	110.2±31.9
				Decrease = improvement: Number of maternal deaths due to postpartum hemorrhages per 1000 women who delivered vaginally in the maternity	122.5±61.1
**Catsambas 2008–2 [52,55,57,63,64]**	Niger	ITS	QIC + training + poster for HCP	% of live births delivered vaginally in the maternity for which immediate breastfeeding within one hour after birth occurred	96.7±2.2
				% of standards observed in essential newborn care among total criteria expected in cases analyzed	85.7±24.9
				% of vaginal deliveries performed in the maternity where the three elements of active management of third stage of labor were applied	89.6±29.6
				% of newborns whose temperature was measured	96.5±2.9
				Decrease = improvement: number of stillbirths per 1000 births in maternity (vaginal and cesarean)	32.3±210.7
				Decrease = improvement: Number of neonatal deaths by time of discharge from hospital per 1000 children born at home or in the maternity (vaginal and cesarean)	221.9±227.4
**Catsambas 2008–3 [52,55,57,63,64]**	Niger	ITS	QIC + training + poster for HCP	% of pre-eclampsia and eclampsia case management criteria that were followed	35.3±10.3
**Westercamp 2017 [65–67]**	Uganda	ITS	QIC + training + patient recording form	% of all-field completeness (number of malaria records with all fields complete/number of malaria records)	60.1±6.7
				% of records with clinically relevant fields completed (number of malaria records with clinically-relevant fields complete/number of malaria records)	61.6±2.6
				% of discordance in malaria case reporting (number of cases in outpatient registry minus number reported in monthly report divided by number of cases in registry)	47.4±66.1
				% of discordance in test-positivity rate reporting (test-positivity rate in lab register minus test-positivity rate in report divided by test-positivity rate of lab register)	57.4±24.5
**Horwood 2017 [68–69]**	South Africa	CBA (RC)	QIC + training + other printed job aid (predominantly focused on LHW)	% of mothers who attended postnatal care within 7 days of delivery at HF	-0.9±4.3
				% of women who reported exclusive breastfeeding for first 6 weeks after birth	18.7±6.0
				% of women who reported being visited by HCP in the first month after birth	45.8±5.8
				% of women who reported being visited by HCP during pregnancy	55.1±5.7
**Webster 2012 [70]**	South Africa	ITS	QIC + training + HCP deployment	Monthly Highly Active Antiretroviral Treatment Initiations (number of HIV positive patients who needed and were initiated on Highly Active Antiretroviral Treatment)	101.1±21
**Waiswa 2017–2 [71–76]**	Uganda	CBA (NRC)	QIC + health services performance reporting + community scorecard	% of women who reported delivering at a HF during their most recent pregnancy (within the past 12 months)	-3.0±6.1
				% of births in which a uterotonic was administered within 1 minute of delivery	8.0±0.8
				% of women who reported immediate breastfeeding within 1 hour of delivery during most recent pregnancy (within past 12 months)	-6.0±5.6
				% of women who knew all three critical danger signs in pregnancy reported during most recent pregnancy (within past 12 months)	-2.0±6.4
**Waiswa 2017–1 [71–78]**	Tanzania	CBA (NRC)	QIC + health services performance reporting + community scorecard	% of women who reported delivering at a HF during their most recent pregnancy (within the past 12 months)	7.0±7.1
				% of births in which a uterotonic was administered within 1 minute of delivery	26.0±0.8
				% of women who reported immediate breastfeeding within 1 hour of delivery during most recent pregnancy (within past 12 months)	-7.0±7.1
				% of women who knew all three critical danger signs in pregnancy reported during most recent pregnancy (within past 12 months)	4.0±7.4
**Colbourn 2013 [42–44,79]**^a^	Malawi	CBA (RC)	QIC + training + group process HCP community + non-medical commodity supply + non-performance- financial incentive + printed materials for HCP + supervision	Maternal mortality rate per 100000 livebirths	-7.6^b^
				Neonatal mortality rate per 1000 livebirths	0.1^b^
				Perinatal mortality rate per 1000 births (stillbirths and early neonatal deaths)	14.2^b^

**CBA (NRC):** Controlled Before-After study with non-randomized controls; **CBA (RC):** Pre-post study with randomized controls; **CITS:** Controlled interrupted time series (with non-randomized controls); **HCP:** Health care provider; **HF:** Health facility; **ITS:** Interrupted time series; **LHW:** Lay or community health workers; **POS-CRT:** Post-only study-cluster randomized trial; **QIC:** Quality improvement collaborative.

^a^ Colbourn 2013 is presented in two rows to indicate two different interventions from the same study.

^b^ Standard error not available.

**Fig 2:**
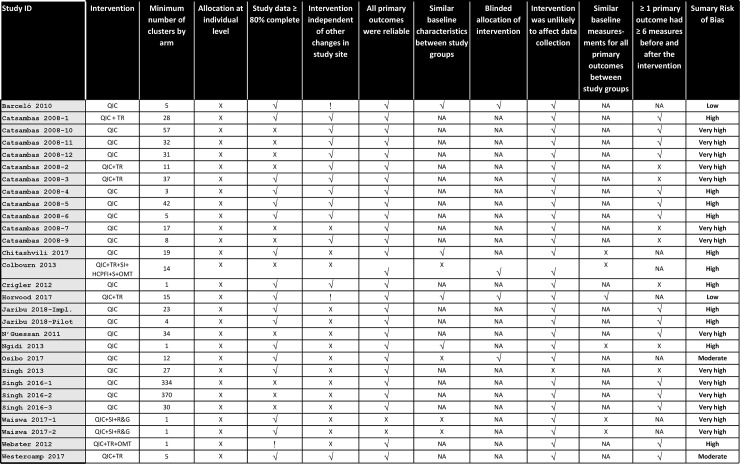
Risk of bias of included studies: Summary and by domain item. √ Yes/done; Unclear; X No/not done; NA Not Applicable. **CBA (NRC):** Controlled Before-After study with non-randomized controls; **CBA (RC):** Pre-post study with randomized controls; **CITS:** Controlled interrupted time series (with non-randomized controls); **HCPFI:** Health Care Professional-directed financial incentives; **ITS:** Interrupted time series; **OMT:** Other management techniques; **POS-CRT:** Post-only study-Cluster randomized trial; **QIC:** Quality Improvement Collaborative; **R&G:** Regulation and governance; **S:** Supervision; **SI:** Strengthening infrastructure; **TR:** Training.

Fig 2 presents the RoB of included studies individually by specific domains. Most studies (25/29, 86.2%) had a high or very high RoB. Two studies had a moderate RoB and two had a low RoB. The 30 study comparisons from 29 studies tested six different strategies that included QICs (Table 2). The most commonly tested QIC intervention had no additional strategy components (21 study comparisons). Other QIC interventions that were tested usually combined QIC with training, with or without additional components. The median study follow-up time was about one year.

**Table 2:** Number of comparisons and risk of bias by quality improvement collaborative strategy.

Strategy	No. of comparisons	Median follow-up time (months)	HCP type	Risk of bias distribution
QIC only (no other strategy components)	21	11.1	HF-based HCPs	1 low, 1 moderate, 9 high, 10 very high
QIC + training	4	8.9	HF-based HCPs	1 moderate, 1 high, 2 very high
QIC + strengthening infrastructure^**a**^ + regulation and governance^**b**^	2	11.0	HF-based HCPs	2 very high
QIC + training + other management techniques^**c**^	1	13.3	HF-based HCPs	1 high
QIC + training + strengthening infrastructure^**d**^ + supervision + other techniques^**e**^	1	13.5	HF-based HCPs	1 high
QIC + training	1	14.5	LHWs	1 low
**Total**	**30**			

**HCP** = Health care provider, **HF** = health facility, **LHW** = lay or community health workers, **QIC** = quality improvement collaborative.

^a^ Report cards (based on household and HF surveys) that summarized data on maternal and newborn health given to HFs and health managers.

^b^ Community scorecard to improve accountability.

^c^ Reorganization of HCP deployment (HCPs rotated to high-volume HFs when high staff turnover and absenteeism were affecting patient care).

^d^ Non-medical commodity supply (bicycles for group facilitators who worked with the community).

^e^ Group process between HCP and community.

In our assessment of publication bias, no strategy-outcome group had the minimum of 10 studies. However, for the one strategy-outcome group with the most studies (QIC intervention, health worker practice outcomes expressed as a percentage, n = 9 studies), the funnel plot revealed no evidence of asymmetry (S2 File).

### Effect of interventions

The findings are summarized in Table 3, which presents QIC intervention effectiveness in terms of median MES (left column) and mean MES (right column and S2 File) from the random effects meta-analysis. Individual effect sizes are presented in Table 1. We had five main findings. First, for the “QIC only” strategy, effectiveness varied highly across outcome groups. For patient behaviors not related to care-seeking, the effect was moderate (median MES: 17.6 percentage points) (Table 3, row 3). For patient health outcomes, there was essentially no effect (0.3 and 1.4 percentage points for percentage and continuous outcomes, respectively). The results ranged from modestly to highly effective for health worker practice outcomes (30.2 to 44.2 percentage points) and patient care-seeking outcomes (7.7 to 62.2 percentage points).

**Table 3:** Summary of findings.

**Population:** Multidisciplinary healthcare teams (and patients that they care for) **Settings:** Health facilities or communities in LMICs **Intervention:** Quality Improvement Collaborative / additional strategies—**Comparison:** Usual care						
Outcomes	**Median MES** (IQR / range)		No. of studies /comparisons (No. of effect sizes)	GRADE ^a^ Certainty of the evidence		Mean MES from random effects meta-analysis (95% CI; No. of comparisons; I^2^)^e^
**Quality improvement collaborative only** (HF-based HCPs; median follow-up 11.5 months)						
**Patient health outcomes**	%^f^	**0.3** (no IQR / -15.0 to 11.4)	3 / 3 (5)	Low^b^	**-2.7** (-16.4 to 10.9; 3; 0%)	
	Cont.^f^	**1.4** (no IQR / -75.3 to 3.0)	3 / 3 (6)^h^		Non-evaluable	
**Patient behaviors related to care-seeking**	%	**7.7** (3.9 to 15.9 / -0.5 to 28.9)	8 / 8 (15)	Low^b^	**5.9** (1.4 to 10.4; 8; 0%)	
	Cont.	**62.2** (20.4 to 85.7 / 7.1 to 116.1)	6 / 6 (7)		**17.6** (-5.9 to 41.4; 6; 0%)	
**Patient behaviors not related to care-seeking^g^**	%	**17.6** (no IQR / 12.5 to 26.2)	3 / 3 (3)	Very low ^b^^,^^c^	**16.0** (9.9 to 22.1; 3; 59%)	
**Health worker practice outcomes**	%	**30.2** (19.6 to 49.7 / 8.1 to 71.2)	9 / 9 (19)	Very low^b^^,^^d^ Low^b^	**36.3** (22.5 to 50.2; 9; 61%)	
	Cont.	**44.2** (no IQR / -28.2 to 135.2)	4 / 4 (6)		**4.2** (-3.7 to 12.1; 3; 0%)	
**Facilitators (e.g., % of HW with job description)**	%	**47.2** (no IQR or range)	1 / 1 (1)	Low ^b^	Non-evaluable	
**Quality improvement collaborative + training** (HF-based HCPs; median follow-up 8.9 months)						
**Patient health outcomes**	Cont.	**111.6** (no IQR / 96.0 to 127.1)	2 / 2 (7)	Low^b^	**96.4** (51.6 to 141.3; 2; 0%)	
**Patient behaviors not related to care-seeking^g^**	%	**87.7** (no IQR / 78.7 to 96.7)	2 / 2 (2)	Very low^b^^,^^d^	**88.0** (70.3 to 105.6; 2; 94%)	
**Health worker practice outcomes**	%	**63.4** (no IQR / 35.3 to 89.6)	4 / 4 (10)	Very low^b^^,^^d^	**60.9** (48.4 to 73.3; 4; 72%)	
	Cont.	**52.4** (no IQR or range)	1 / 1 (2)		Non-evaluable	
**Quality improvement collaborative + training + other management techniques** (HF-based HCPs; median follow-up 13.3 months)						
**Patient behaviors related to care-seeking**	Cont.	**101.1** (no IQR or range)	1 / 1 (1)	Low^b^	Non-evaluable	
**Quality improvement collaborative + training + strengthen infrastructure + supervision + other management techniques** (HF-based HCPs; median follow-up 13.5 months)						
**Patient health outcomes**	Cont.	**0.1** (no IQR or range)	1 / 1 (3)^i^	Very low ^b^	Non-evaluable	
**Quality improvement collaborative + strengthen infrastructure + regulation and governance** (HF-based HCPs; median follow-up 11.0 months)						
**Patient behaviors related to care-seeking**	%	**9.5** (no IQR / 2.5 to 16.5)	2 / 2 (4)^i^	Very low^b^^,^^d^	**9.3** (-4.4 to 23.0; 2; 82%)	
**Patient behaviors not related to care-seeking^g^**	%	**-2.8** (no IQR / -4.0 to -1.5)	2 / 2 (4)^i^	Very low ^b^	**-3.0** (-11.0 to 5.1; 2; 0%)	
**Quality improvement collaborative + training** (focused predominantly on lay/community health workers; median follow-up 14.4 months)						
**Patient behaviors related to care-seeking**	%	**-0.9** (no IQR or range)	1 / 1 (1)^i^	Low	Non-evaluable	
**Patient behaviors not related to care-seeking^f^**	%	**18.7** (no IQR or range)	1 / 1 (1)^i^	Low	Non-evaluable	
**Health worker practice outcomes**	%	**50.4** (no IQR or range)	1 / 1 (2)^i^	Moderate	Non-evaluable	

**MES:** median effect size per comparison; **95% CI:** 95% confidence interval; **HF-based HCPs:** health facility-based health care providers; **IQR:** Interquartile range

^a^ GRADE: The certainty evidence for RCTs, ITS studies, and other non-randomized studies started at high, moderate, and low, respectively.

High certainty: Very good indication of the likely effect. The likelihood that the effect will be substantially different is low.

Moderate certainty: Good indication of the likely effect. The likelihood that the effect will be substantially different is moderate.

Low certainty: Some indication of the likely effect. However, the likelihood that it will be substantially different is high.

Very low certainty: Not a reliable indication of the likely effect. The likelihood that the effect will be markedly different is very high.

^b^ Certainty evidence was downgraded 1 level for serious risk of bias.

^c^ Certainty evidence was downgraded 1 level for serious inconsistency.

^d^ Certainty evidence was downgraded 2 levels for very serious inconsistency.

^e^ Meta-analysis could only be performed if the number of median effect sizes was > 1 and their standard errors were available.

^f^ %: outcome expressed as a percentage, Cont.: outcome expressed as continuous and unbounded.

^g^ For example, patient adherence to treatment regimen.

^h^ Three out of 6 effect sizes were from controlled before-after study.

^i^ All effect sizes from were controlled before-after studies.

Second, for the “QCI + training” strategy for health facility-based HCPs, although there were only 4 studies, effectiveness was very high: MES 52.4 to 63.4 percentage points for health worker practice outcomes, 111.6 percentage points for patient health outcomes, and 87.7 percentage points for non-care-seeking patient behaviors (Table 3, rows 6–8). An additional study on a similar strategy (QIC + training + other management techniques) also found very high effectiveness (101.1 percentage points) for its one outcome on care-seeking patient behaviors.

Third, for the “QIC + training + strengthening infrastructure (bicycles for facilitators) + supervision + other management techniques (group process between HCP and community)” strategy, the one study found essentially no effect (MES 0.1 percentage points, for patient health outcomes) (Table 3, row 10). Fourth, for the “QIC + strengthening infrastructure (report cards) + regulation and governance (community scorecards)” strategy, the effectiveness from two studies ranged from essentially no effect (-2.8 percentage points, for non-care-seeking patient behaviors) to modest effect (9.5 percentage points, for care-seeking patient behaviors) (Table 3, rows 11–12).

Finally, the one study of lay health workers found highly variable results, ranging from essentially no effect (-0.9 percentage points, for care-seeking patient behaviors) to moderately large effects (18.7 percentage points, for non-care-seeking patient behaviors) to very large effects (50.4 percentage points, for health worker practice outcomes) (Table 3, rows 13–15).

Both the random effects meta-analysis considering one median effect size per study comparison for each outcome (Table 3), and the sensitivity analysis considering all effect sizes individually (S3 File) were consistent with the primary analysis. The certainty of the evidence according to GRADE criteria was low or very low for all strategy-outcome combinations, except for the effect of QIC + training on health worker practice outcomes for lay health workers (moderate certainty). However, as the result for this last group is based on only a single study, the generalizability is extremely limited.

## Discussion

This systematic review and meta-analysis on QICs in LMICs showed variable effectiveness across different outcomes and strategies. The quality of the evidence was mainly low or very low [17]. We found consistent results using different statistical approaches.

In summary, among studies of health facility-based HCPs, for the “QIC only” strategy, effectiveness varied highly across outcome groups, with no effect for patient health outcomes. For the “QIC + training” strategy, effectiveness might be very high for patient health outcomes, HCP practice outcomes, and care-seeking. Adding other management techniques to this strategy might also be highly effective for patient care-seeking behaviors. The effect of “QIC + training + strengthening infrastructure + supervision + other management techniques” or “QIC + strengthening infrastructure + regulation and governance” strategies seemed small to modest.

The only study assessing lay health workers showed effects that varied from essentially no effect on care-seeking patient behaviors to a large effect on non-care-seeking patient behaviors and HCP practice outcomes.

The main limitations of our systematic review were low quality of the evidence, scarce data on long-term effects, and heterogeneous outcomes. Also, some included studies came from unpublished gray literature, and several were conducted by the same group of authors. We attempted to address any potential imbalance in the quality of these studies by applying the same risk-of-bias assessment to all included studies. Furthermore, the random effects meta-analysis in this review was limited by the low quality of studies and wide diversity of outcomes. However, we believe meta-analysis as a secondary analysis tool provided useful complemental information about the direction, magnitude, and precision of intervention effects. Strengths of our review were that it was based on an extensive literature review from multiple sources, it used a single analytic framework with comparable effect sizes (as opposed to reporting different effect sizes, such as odds ratios and risk differences, from different studies), and it focused on LMIC settings. Its results can inform decision-making for health programs and intervention implementers with regards to which QIC-based interventions are most effective for improving which aspects of health systems in LMICs. Considering the small number of studies for each main comparison and the low quality of evidence, this review also highlights substantial evidence gaps and important opportunities for improvement in the conduct of future QIC studies.

Previous systematic reviews have approached the topic of QIC effectiveness in different ways and did not include several studies captured by our work [8–10]; nevertheless, they found similar effects and evidence gaps. Numerous potential determinants of QIC success were evaluated in a systematic review that did not include any of the primary studies included in our review, and only a few related to empirical effectiveness [24]. For example, some aspects of teamwork and participation in specific collaborative activities seem to improve short-term success, while sustainability of teams and continued data gathering enhanced the chances of long-term success. In a study currently underway, the impact of district-led learning on clinical practice and patient outcomes, communication, HCP motivation, and team dynamics are being explored [25, 26]. It would be desirable for future studies to examine what core components of QICs are related to patient- and provider-level outcomes.

Our findings clearly show that there is still not a solid evidence base on the effect of QICs in LMICs, although our results suggest that there are situations in which QICs could be considered. QICs are not static structures–rather, they have been implemented and adapted in a number of ways to achieve their stated aims. Some common adaptations include their use for generating new ideas and for empowering HCPs. Although based on relatively few studies, our review’s results suggest that combining QICs with training might be the most effective approach for implementing QICs.

Finally, on the recommendation for additional studies on QICs, we think that the ideal study design would be an interrupted time series with a randomized control group. The justification is that such a design would allow for an overall evaluation of intervention effectiveness as well as an evaluation of heterogeneity of effectiveness among sites. The design would also allow for a characterization of the effect over time. Other attributes include a follow-up time of at least 12 months, an objective data source for the evaluation (i.e., not only data collected by the QI teams unless the data quality is reasonably good and data quality does not change over time), a sample size that reflects real-world QICs (i.e., at least 20 facilities per study arm), qualitative and process evaluation components to describe how the intervention worked, a costing and economic evaluation, and an assessment of whether the intervention had any negative effects (e.g., drawing health workers’ attention to one aspect of care that decreases quality for other aspects of care).

In conclusion, the overall quality of the evidence on the effectiveness of QICs in LMICs was low. Based on the large and variable effect sizes seen in some outcome groups, additional research with high-quality studies is warranted to provide a more reliable and precise estimation of the effect of this promising intervention.

## Supporting information

S1 Checklist PRISMA checklist. (PDF)

S1 File Details of the search strategy. (PDF)

S2 File Meta-analysis results, forest plots, and funnel plots. (PDF)

S3 File Sensitivity analysis and list of excluded studies. (PDF)
