# Supplementary material for: The effectiveness of the quality improvement collaborative strategy in low- and middle-income countries: A systematic review and meta-analysis
Source: PLoS One. 2019 Oct 3;14(10):e0221919. doi: 10.1371/journal.pone.0221919 (PMC6776335; doi:10.1371/journal.pone.0221919)
Supplement: S1 File — (PDF) [file pone.0221919.s002.pdf]

ANNEX 1. Details of the search strategy and reference of included studies

Section A. Summary

The literature search was conducted in two phases. First, we searched results of the Health Care Provider Performance Review (HCPPR), which is a comprehensive systematic review of the effectiveness of strategies to improve health worker performance in low- and middle-income countries (see Section B for details). Second, we updated the literature search with a focus on studies of collaborative improvement (see Section C for details).

Section B. First phase of the literature search

For the HCPPR, two literature searches were conducted: the original search (conducted from 2006–2008) and an updated search (conducted from October 2015–May 2016). Details of the original search are presented in Section B1, and details of the updated search are presented in Section B2.

Section B1. Methods of the original HCPPR search

The literature search strategy had six components. First, we searched 15 electronic databases (Box 1). The search strategy was based on that used by the International Network for the Rational Use of Drugs (INRUD). These databases were searched in groups on May 2006, September 2006, and May 2007. All searches went back in time as far as the databases allowed. Second, we searched our personal libraries and asked eleven colleagues for references and unpublished studies. Third, we searched document inventories and websites of 30 organizations involved with health worker performance (Box 1). This component of the search was done between January 2006 and April 2010. Fourth, we performed a hand search of bibliographies from 510 previous reviews and other articles. Fifth, after being contacted to answer questions concerning their studies, 17 authors of studies that were included in the review sent additional, new reports related to their studies. Sixth, after reading an included report that lacked many basic details (e.g., a short presentation at a scientific conference), data abstractors searched the Internet for supplemental articles that could be abstracted along with that report. Additional details of the original search are presented in Rowe SY *et al*., in PLOS ONE.[1]

---

[1] Rowe SY, Peters DH, Holloway KA, Chalker J, Ross-Degnan D, Rowe AK (2019). A systematic review of the effectiveness of strategies to improve health care provider performance in low- and middle-income countries: Methods and descriptive results of included studies. PLoS ONE 14(5): e0217617.https://doi.org/10.1371/journal.pone.0217617.

Box 1. Details of the original HCPPR literature search

Electronic databases searched

Campbell Collaboration, Cumulative Index to Nursing & Allied Health Literature (CINAHL), Cochrane Library (which includes the Database of Abstracts of Review of Effects [DARE] and the Cochrane Central Register of Controlled Trials [CENTRAL]), Dissertation Abstracts (for theses and dissertations), EconLit, Eldis, EMBASE, the Effective Practice and Organisation of Care (EPOC) specialized register, Education Resources Information Center (ERIC), Global Health, The Healthcare Management Information Consortium (HMIC), MEDLINE, Science Citation Index (SCI), Sociological Abstracts, and Social Sciences Citation Index (SSCI).

Document inventories and websites searched

Basic Support for Institutionalizing Child Survival (BASICS); Capacity Project; U.S. Centers for Disease Control and Prevention; Center for Global Development; CORE group; Danish International Development Agency; U.K. Department for International Development; EngenderHealth; Global Alliance for Vaccines and Immunization; Global Fund to Fight AIDS, Tuberculosis, and Malaria; HealthNet TPO; Human Resources for Health Resource Center; International Conference on Social Health Insurance in Developing Countries (Berlin, December 2005); International Conference on Improving Use of Medicines (ICIUM) 1997 and 2004 conference proceedings; Institute for Healthcare Improvement; WHO/INRUD database [50]; JHPIEGO; Management Sciences for Health; Pan American Health Organization; Partners in Health; PHRPlus; Population Council; PRIME II Project; Partnership for Social Science in Malaria Control; Quality Assurance Project; Safe Injection Global Network; United Nations Children's Fund (UNICEF); U.S. Agency for International Development (USAID DEC); World Bank; and WHO.

The figure below summarizes the results of the original HCPPR literature search.

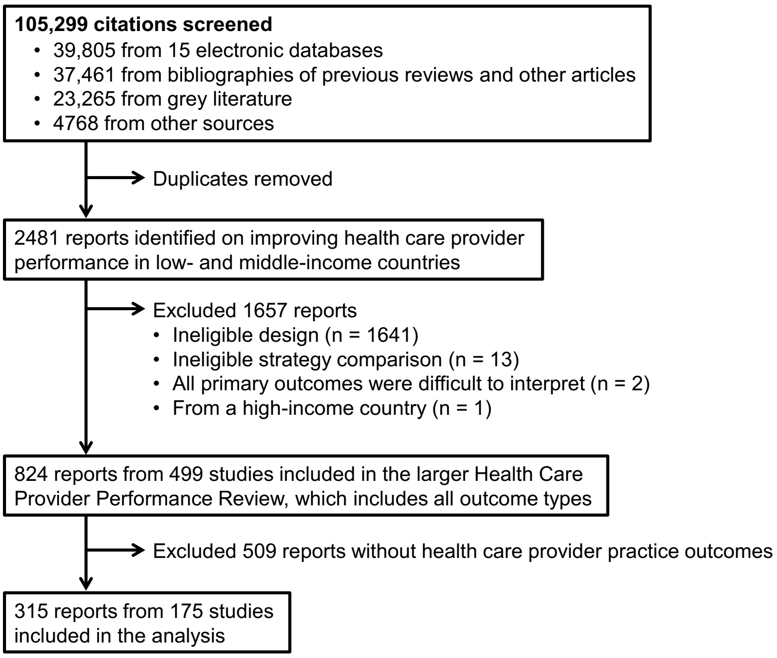

Section B2. Methods of the updated HCPPR literature search

The methods for the updated literature search were based on those used in the original version of the HCPPR and guidance from the Cochrane Effective Practice and Organisation of Care study group (EPOC) (EPOC, 2013). Most of the updated search was conducted between October 2015 and May 2016. Some initial searching for unpublished studies was done from March–April 2014 and from June–July 2015. The updated HCPPR literature search had the following 11 components:

1. We searched 16 electronic databases of published studies by: a) applying a filter of search terms to obtain a subset of citations that would likely include eligible studies (Box 2), b) ranking the resulting citations with a computer algorithm developed by the Johns Hopkins University Applied Physics Laboratory (JHU-APL), and c) screening the top 3600 citations:
   - AfricaBib databases: Africana Periodical Literature and African Women and Kenya Coast
   - BLDS British Library for Development Studies
   - CINAHL: Cumulative Index to Nursing & Allied Health Literature
   - Cochrane Library: Cochrane Central Register of Controlled Trials (CENTRAL), Cochrane Reviews, Economic Evaluations, Methods Studies, Other Reviews, Technology Assessments
   - CRD: Center for Reviews and Dissemination
   - EconLit
   - EPPI DoPHER: EPPI-Database of promoting health effectiveness reviews
   - EPPI TroPHI: EPPI-Trials Register of Promoting Health Interventions
   - ICTRP: International Clinical Trials Registry Platform
   - INRUD Medicines Use Bibliography
   - JOLIS library catalogue - International Monetary Fund, World Bank and International Finance Corporation
   - POPLINE
   - PubMed/MEDLINE
   - SCOPUS
   - Sociological Abstracts
   - WPRIM: Index Medicus for the Western Pacific

2. We searched 34 electronic databases of published studies (two of which were the same as the 16 databases described above in component 1) by: a) applying a filter of search terms to obtain a subset of citations that would like include eligible studies, and b) screening all the resulting citations. This group of databases was not ranked by the JHU-APL algorithm because the JHU-APL staff could not process the databases, given the available resources.
   - 3ie
   - AFROLIB Database
   - AIM: African Index Medicus
   - AJOL: African Journals Online
   - BanglaJOL: Bangladesh Journals Online
   - Bibliomap: EPPI-Centre database of health promotion research
   - BVSCuba (Biblioteca Virtual En Salud De Cuba)/InfoMed
   - Campbell Collaboration
   - DOAJ

- EAKN: EurasiaHealth AIDS Knowledge Network
- East View Information Service Online Databases
- ELDIS
- Embase
- EPPI-Centre reviews in health systems and international development
- EPPI-Trophi
- ERIC
- Global Health and Global Health Archive
- HERDIN
- HINARI: Health InterNetwork Access to Research Initiative
- ICI: Indian Citation Index
- IDEAS Economics and Finance database (RePEc)
- IndMED
- Indonesian Publication Index
- JOLIS
- LAMJOL: Latin American Journals Online
- NepJOL: Nepal Journals Online
- Pakmedinet
- PhilJOL: Phillipines Journal Online
- PLOS One
- ProQuest Central
- SOURCE: International online resource centre on disability and inclusion
- USAID mHealth Compendium Database
- WHO-INRUD Medicines Use Database
- WHOLIS

3. We assessed 34 electronic databases of published studies that were recommended by EPOC[2] (EPOC, 2013) (that were different from the 47 databases described above in components 1 and 2), but we decided not to search them because either: a) the database included studies primarily from a high-income country (e.g., Greece), b) studies in the database were in another database already searched, c) the database required a paid subscription and included few studies from LMICs, or d) the database (including the database's search engine) were in language that was difficult for the HCPPR Team to translate (e.g., Turkish or Vietnamese).
    - BabelMeSH
    - Biomedicina Croatica
    - Chinese Medicine Premier (Wanfang Data)
    - Chinese Scientific Journal Database
    - Collaboration for Evidence Based Healthcare in Africa (CEBHA)
    - Cochrane Library Cochrane Groups

---

[2] Effective Practice and Organisation of Care (EPOC). A collection of databases, web sites and journals relevant to Low- and Middle-Income Countries (LMICs). Oslo: Norwegian Knowledge Centre for the Health Services; August 2013. Available at Internet address: http://epoc.cochrane.org/sites/epoc.cochrane.org/files/uploads/LMIC%20Databases%20August%202013.pdf, accessed September 15, 2015.

- Dissertation Abstracts (now called ProQuest Dissertation & Theses Global (PQDT Global))
- EPOC Register
- EPOC Reviews
- Essential Health Links
- Global Health Gateway
- Global Health Library (GHL) of WHO
- Hellenic Ph.D. Dissertations Thesis
- HMIC
- Hrcak
- IranMedex
- KoreaMed
- Latindex
- LILACS
- Magyar Orvosi Bibliográfia (Bibliographia Medica Hungarica)
- MedCarib
- Medical Bibliography – Hippocrates
- Medical databases (Russia)
- PAHO Library Catalogue
- Panteleimon
- Psikiyatri Dizini
- Rx for Change
- SciDev Net: Science and Development Network
- Science Citation Index (now Science Citation Index Expanded)
- SSCI: Social Sciences Citation Index
- Turk MEDLINE
- Türk Tıp Veri Tabanı
- University of Zagreb Medical School Repository
- VJOL: Vietnam Journals Online

4. We searched 15 electronic databases of published studies (largely overlapping the databases in component 1, above) by: a) downloading all studies in the database, b) ranking the citations with the JHU-APL computer algorithm, and c) screening the top 2400 citations.
    - AJOL
    - BLDS British Library for Development Studies
    - Campbell Collaboration
    - Center for Reviews and Dissemination
    - Cochrane Library
    - Cumulative Index to Nursing & Allied Health Literature
    - EconLit
    - EMBASE (Jan 2015 sample)
    - EPPI DoPHER
    - EPPI TRoPHI
    - GlobalHealth (2015 sample)
    - INRUD Medicines Use Bibliography
    - JOLIS (2015 sample)
    - POPLINE (2015 sample)

- PubMed/MEDLINE

5. We searched websites of nine scientific conferences and technical meetings to identify unpublished studies.
    - Global Health and Innovation Conference 2015
    - Global Maternal Newborn Health Conference 2013 and 2015
    - Global Symposium on Health Systems Strengthening 2010, 2012, and 2014
    - ICIUM 2011 Conference Proceedings
    - ICSHIDC: International Conference on Social Health Insurance in Developing Countries 2007
    - ISQua (International Society for Quality in Health Care) International Conference 2009-2015
    - JHPIEGO mHealth Summit 2015
    - Maternal Health Task Force Technical Meetings 2011-2014
    - Trop Med (American Society of Tropical Medicine & Hygiene) Conference 2006-2015

6. We searched websites of 44 organizations that work on HCP performance issues to identify unpublished studies.
    - ACT Consortium
    - BBC Media Action
    - Capacity Project
    - CARE Group
    - CDC websites & publications
    - Center for Global Development
    - COMDIS-HSD
    - CORDAID
    - CORE Group
    - D-tree
    - DANIDA
    - DFID
    - Engender Health
    - Gavi, The Vaccine Alliance
    - Global Fund to Fight AIDS, TB, and Malaria
    - Harvard School of Public Health
    - Health Systems Evidence
    - HealthNet TPO online library
    - HPSA Africa
    - Human Resources of Health Resource Center
    - ICCP (International Cancer Control Partnership) Portal
    - IHI: Institute for Healthcare Improvement
    - INRUD
    - JHPIEGO
    - Malaria Consortium
    - Measles and Rubella Initiative
    - mHealth Evidence
    - mpoweringhealth
    - MSH: Management Sciences for Health

- Nuffield Center for International Health
- PAHO
- Partners in Health
- PATH
- PHRPlus
- Population Council
- Respond Project
- Safe Injections Global Network (SIGN)
- STEPS Country Reports
- UNICEF
- USAID
- USAID Assist
- WHO
- World Bank Documents & Reports
- World Bank Open Knowledge Repository

7. We assessed seven other websites, but we decided not to search them either because: the website had not been updated since the literature search for the previous version of the HCPPR, the website was no longer active, or because we lacked the resources to search them.
    - http://www.dktinternational.org/publications-resources
    - http://healthmarketinnovations.org/programs/search
    - Annual Meeting of the Safe Injection Global Network (SIGN) 2007-2010 (e.g., URL for 2010 meeting: http://www.who.int/injection_safety/toolbox/sign2010_meeting.pdf?ua=1)
    - http://www.prime2.org/prime2/section/44.html
    - http://www.malaria.org/PSSMC/publications.html
    - http://www.malaria.org/PSSMC/reports.html
    - http://www.qaproject.org/products.html

8. We contacted 46 experts for study reports, bibliography lists, or website ideas.
    - Smisha Agarwal
    - Ray Arindam
    - Pierre Barker
    - Sebastian Bauhoff
    - Edward Broughton
    - John Chalker
    - Ingrid Chen
    - Mushtque Chowdhury
    - Sian Clarke
    - Valérie D'Acremont
    - Jishnu Das
    - Manuela De Allegri
    - Damien de Walque
    - Clara Delavallade
    - Brian DeRenzi
    - Sabine Gies
    - Christopher Gill

- Nemat Hajeebhoy
- Jim Heiby
- Lisa Hirschhorn
- Kathy Holloway
- Kiersten Israel-Ballard
- Anunaya Jain
- Krishnamurthy Jayanna
- Karin Kallander
- Hnin Su Khin
- Freddy Kitutu
- Christina Marie Braüner Klokkenga
- Alain Labrique
- Sham Lal
- Christopher Lourenco
- Hema Magge
- Manoj Mohanan
- Chris Morgan
- Bright Clement Orji
- Berk Özler
- Henry Perry
- Vikrant Prabhakar
- Clotilde Rambaud-Althaus
- Arindam Ray
- Sreera Sasi
- Freddie Peter Ssengooba
- Sarah Staedke
- May Sudhinaraset
- Jakob Svensonii
- Siddhartha Swarup

9. We screened the bibliographies of 351 review articles on HCP performance that were identified from screening the results of literature search components 1-8.

10. We screened 234 other non-review-article documents that were identified from screening the results of the literature search components 1-8, or identified during the process of abstracting study reports (e.g., reports received from a study author, identified in the main study report's reference list, or identified during an internet search for more information related to a study).

11. We identified 405 potentially relevant review articles, but we decided not to screen their bibliographies because the review's inclusion criteria did not match that of our review (N=61) (e.g., the review only included non-LMIC studies), or because the review articles were published in 2006 or earlier (these "older" reviews likely had titles that we screened already in the original version of the HCPPR) or because we lacked the resources to screen them (N=344).

Box 2. Search strategy for Pubmed

1. exp health personnel/ not ("coroners and medical examiners"/ or veterinarians/)
2. exp Physicians/
3. (physician* or doctor*).mp.
4. exp Nurses/
5. (nurse* or nursing).mp.
6. (clinical officer* or medical officer*).mp.
7. Midwifery/
8. (midwive* or midwifery).mp.
9. Nurses' Aides/
10. (health auxiliar* or health assistant*).mp.
11. Pharmacists/
12. pharmacist*.mp.
13. Medical Laboratory Personnel/
14. (laboratory worker* or laboratory personnel).mp.
15. ((medic* or drug*) adj3 (vendor* or sell*)).mp.
16. shopkeeper*.mp.
17. Community Health Workers/
18. community health worker*.mp.
19. village health worker*.mp.
20. lay health worker*.mp.
21. birth attendant*.mp.
22. women* group*.mp.
23. Health Educators/
24. health educator*.mp.
25. health worker*.mp.
26. exp hospitals/ not Hospitals, Animal/
27. inpatient ward*.mp.
28. inpatient service*.mp.
29. exp Emergency Service, Hospital/
30. emergency department*.mp.
31. Outpatient Clinics, Hospital/
32. outpatient department*.mp.
33. clinic*.mp.
34. exp Health Facilities/
35. health facilit*.mp.
36. health post*.mp.
37. exp Pharmacy/
38. (pharmacy or pharmacies).mp.
39. (drug adj (shop* or store* or kiosk*)).mp.
40. exp Laboratories/
41. laborator*.mp.
42. ("health care provider*" or "healthcare provider*" or "private provider*").mp.
43. or/1-42
44. exp Quality Assurance, Health Care/
45. (quality adj3 care).mp.
46. Employee Performance Appraisal/
47. (employee* adj3 perform*).mp.
48. ((duty or duties) adj3 perform*).mp.

49. (task* adj3 perform*).mp.
50. (work* adj3 perform*).mp.
51. guideline/ or practice guideline/
52. (practice* adj3 guideline*).mp.
53. guidelines as topic/ or practice guidelines as topic/
54. guideline adherence/
55. (guideline* adj3 adherence).mp.
56. clinical competence/
57. clinical* competen*.mp.
58. quality improvement/
59. (quality adj3 improve*).mp.
60. ((guideline* or practice*) adj3 complian*).mp.
61. Patient Compliance/
62. patient* complian*.mp.
63. ("availability of supplies and equipment" or HCP attitudes or HCP knowledge or HCP satisfaction or supervision or assessment or case management or chemoprophylaxis or consultation time or "counseling and communication" or diagnosis or HCP documentation or referral or treatment or vaccination or morbidity or mortality or patient care-seeking or ((patient or caregiver) adj knowledge) or ((patient or community) adj attitude*) or patient satisfaction).mp.
64. or/44-63
65. infrastructure.mp.
66. financing.mp.
67. Motivation/
68. incentive*.mp.
69. government regulation/
70. regulation*.mp.
71. jurisprudence/
72. legislation, drug/
73. "drug and narcotic control"/
74. mandatory reporting/
75. Decision Making, Organizational/
76. governance.mp.
77. "Codes of Ethics"/
78. (code* adj3 ethic*).mp.
79. exp Licensure/
80. licens*.mp.
81. accreditation/
82. accredit*.mp.
83. certification/
84. certification*.mp.
85. "facility regulation and control"/
86. Decision Making/
87. (decision* adj3 making).mp.
88. management.mp.
89. Problem Solving/
90. (problem* adj3 solv*).mp.
91. exp "Organization and Administration"/
92. supervision.mp.
93. benchmarking/

94. benchmark*.mp.
95. "peer review"/ or peer review, health care/
96. (peer* adj3 review*).mp.
97. training.mp.
98. (job adj3 aid*).mp.
99. reminder systems/
100. (remind* adj3 system*).mp.
101. decision support techniques/
102. (decision* adj3 aid*).mp.
103. cell phones/ or text messaging/
104. ((cell* or mobile*) adj3 phone*).mp.
105. (text adj3 messag*).mp.
106. (audit* or Bamako Initiative* or bulletin* or collaborative improvement* or community case management* or committee* or computer or continuous quality improvement* or CQI or contracting* or data collection or drugs or education or "integrated management of childhood illness" or IMCI* or literature or maintenance or medical record* or monitoring or newsletter* or pamphlet* or performance reporting or poster* or problem-solving).mp.
107. (equipment or essential drug* or feedback* or fees or funds or group meeting or group process* or improvement collaborative* or insurance or integrate or integration or quality improvement* or recognition or registration or reimbursement or repair or standard* or system or technology or total quality management* or TQM* or assessment*).mp.
108. (scorecard* or dashboard* or "pay for performance" or detailing).mp.
109. or/65-108
110. Developing Countries/
111. (Africa or Asia or Caribbean or West Indies or South America or Latin America or Central America).mp.
112. (Afghanistan or Albania or Algeria or Angola or Armenia or Armenian or Azerbaijan or Bangladesh or Benin or Byelarus or Byelorussian or Belarus or Belorussian or Belorussia or Belize or Bhutan or Bolivia or Bosnia or Herzegovina or Hercegovina or Botswana or Brasil or Brazil or Bulgaria or Burkina Faso or Burkina Fasso or Upper Volta or Burundi or Urundi or Cambodia or Khmer Republic or Kampuchea or Cameroon or Cameroons or Cameron or Camerons or Cape Verde or Central African Republic or Chad or China or Colombia or Comoros or Comoro Islands or Comores or Mayotte or Congo or Zaire or Costa Rica or Cote d'Ivoire or Ivory Coast or Cuba or Djibouti or French Somaliland or Dominica or Dominican Republic or East Timor or East Timur or Timor Leste or Ecuador or Egypt or United Arab Republic or El Salvador or Eritrea or Ethiopia or Fiji or Gabon or Gabonese Republic or Gambia or Gaza or Georgia Republic or Georgian Republic or Ghana or Gold Coast or Grenada or Guatemala or Guinea or Guiana or Guyana or Haiti or Honduras or India or Maldives or Indonesia or Iran or Iraq or Jamaica or Jordan or Kazakhstan or Kazakh or Kenya or Kiribati or Korea or Kosovo or Kyrgyzstan or Kirghizia or Kyrgyz Republic or Kirghiz or Kirgizstan or Lao PDR or Laos or Lebanon or Lesotho or Basutoland or Liberia or Libya or Macedonia or Madagascar or Malagasy Republic or Malaysia or Malaya or Malay or Sabah or Sarawak or Malawi or Nyasaland or Mali or Marshall Islands or Mauritania or Mauritius or Agalega Islands or Mexico or Micronesia or Middle East or Moldova or Moldovia or Moldovian or Mongolia or Montenegro or Morocco or Ifni or Mozambique or Myanmar or Myanma or Burma or Namibia or Nepal or Nicaragua or Niger or Nigeria or Pakistan or Palau or Palestine or Panama or Paraguay or Peru or Philippines or Philipines or Phillipines or Phillippines or Romania or Rumania or Roumania or Rwanda or Ruanda or Saint Lucia or St Lucia or

Saint Vincent or St Vincent or Grenadines or Samoa or Samoan Islands or Navigator Island or Navigator Islands or Sao Tome or Senegal or Serbia or Montenegro or Sierra Leone or Sri Lanka or Ceylon or Solomon Islands or Somalia or South Africa or Sudan or Suriname or Surinam or Swaziland or Syria or Tajikistan or Tadzhikistan or Tadjikistan or Tadzhik or Tanzania or Thailand or Togo or Togolese Republic or Tonga or Tunisia or Turkey or Turkmenistan or Turkmen or Uganda or Ukraine or USSR or Soviet Union or Union of Soviet Socialist Republics or Uzbekistan or Uzbek or Vanuatu or New Hebrides or Vietnam or Viet Nam or West Bank or Yemen or Zambia or Zimbabwe or Rhodesia).mp.
113. ((developing or less* developed or under developed or underdeveloped or middle income or low* income or underserved or under served or deprived or poor*) adj (countr* or nation? or population? or world)).ti,ab.
114. ((developing or less* developed or under developed or underdeveloped or middle income or low* income) adj (economy or economies)).ti,ab.
115. (low* adj (gdp or gnp or gross domestic or gross national)).ti,ab.
116. (low adj3 middle adj3 countr*).ti,ab.
117. (lmic or lmics or third world or lami countr*).ti,ab.
118. transitional countr*.ti,ab.
119. or/110-118
120. randomized controlled trial.pt.
121. controlled clinical trial.pt.
122. multicenter study.pt.
123. (randomis* or randomiz* or randomly allocat* or random allocat*).ti,ab.
124. groups.ab.
125. (trial or multicenter or multi center or multicentre or multi centre).ti.
126. (intervention* or controlled or control group or compare or compared or (before adj5 after) or (pre adj5 post) or pretest or pre test or posttest or post test or quasiexperiment* or quasi experiment* or evaluat* or effect or impact or time series or time point? or repeated measur*).ti,ab.
127. or/120-126
128. exp Animals/
129. Humans/
130. 128 not (128 and 129)
131. news.pt.
132. comment.pt.
133. editorial.pt.
134. comment on.cm.
135. or/130-134
136. 127 not 135
137. 43 and 64 and 109 and 119 and 136
138. limit 137 to yr="2006 -Current"
139. 43 and 64 and 109 and 119
140. 139 not 137
141. limit 140 to yr="2006 -Current"
142. 43 and 64 and 109 and 136 and (Western Sahara or Nauru or Tuvalu).mp.
143. 142 not (138 or 141)

The table below summarizes the title screening and full text screening results for the 11 components of the updated HCPPR literature search.

	Component	Eligibility filter applied	Ranked by JHU-APL	No. title collections	No. titles screened	No. full text screened
1	Electronic databases of published studies	✓	✓	16	3600	1290
2	Electronic databases of published studies	✓	×	34	12919	1180
3	Electronic databases of published studies not searched	×	×	34	0	0
4	Electronic databases of published studies	×	✓	15	2400	786
5	Websites of scientific conferences or technical meetings	✓	×	9	24036	1059
6	Websites of organizations that work on HCP performance	✓	×	44	44646	1135
7	Websites not searched	×	×	7	0	0
8	Documents from experts	✓	×	46	285	285
9	Bibliographies of review articles	✓	×	351	23058	2248
10	Other documents	✓	×	115	234	234
11	Review articles whose reference lists were not screened	✓	×	405	0	0
	**Total**			**1076**	**111178**	**8217**

Section C. Second phase of the literature search. Update search focused on collaborative improvement. ***Search Date March 15, 2019.***

PubMed

Search	Query
#23	Search **(#5 OR #21)** Filters: **Publication date from 2015/09/24**
#22	Search **(#5 OR #21)**
#21	Search **(#20 AND #10 AND #14 AND #19)**
#20	Search **(#6 OR #7 OR #8 OR #9)**
#19	Search **(#15 OR #16 OR #17 OR #18)**
#18	Search **Care Research[tiab]**
#17	Search **Healthcare Research[tiab]**
#16	Search **Health Services Evaluat*[tiab]**
#15	Search **Health Services Research[Mesh]**
#14	Search **(#11 OR #12 OR #13)**
#13	Search **Quality Assurance[tiab]**
#12	Search **Program Evaluat*[tiab]**
#11	Search **Total Quality Management[Mesh]**
#10	Search **("Outcome and Process Assessment (Health Care)"[Mesh])**
#9	Search **Cooperative Behav*[tiab]**
#8	Search **Organisational Model*[tiab]**
#7	Search **Organizational Model*[tiab]**
#6	Search **Organizational Innovation[Mesh]**
#5	Search **(#3 AND #4)**
#4	Search **Collaborative*[tiab]**
#3	Search **(#1 OR #2)**
#2	Search **Quality Improve*[tiab]**
#1	Search **Quality Improvement[Mesh]**

Cochrane Library (Wiley) (n=**36**)

ID	Search
#1	MeSH descriptor: [Quality Improvement] explode all trees
#2	Quality Improve*:ti,ab,kw (Word variations have been searched)
#3	#1 or #2
#4	Collaborative*:ti,ab,kw (Word variations have been searched)
#5	#3 and #4
#6	MeSH descriptor: [Organizational Innovation] explode all trees
#7	Organizational Model*:ti,ab,kw (Word variations have been searched)
#8	Organisational Model*:ti,ab,kw (Word variations have been searched)
#9	Cooperative Behav*:ti,ab,kw (Word variations have been searched)
#10	#6 or #7 or #8 or #9
#11	MeSH descriptor: [Outcome and Process Assessment (Health Care)] explode all trees
#12	MeSH descriptor: [Total Quality Management] explode all trees
#13	Program Evaluat*:ti,ab,kw (Word variations have been searched)
#14	Quality Assurance:ti,ab,kw (Word variations have been searched)

#15 #11 or #12 or #13 or #14
#16 MeSH descriptor: [Health Services Research] explode all trees
#17 Health Services Evaluat*:ti,ab,kw (Word variations have been searched)
#18 Healthcare Research:ti,ab,kw (Word variations have been searched)
#19 Care Research:ti,ab,kw (Word variations have been searched)
#20 #16 or #17 or #18 or #19
#21 #10 and #11 and #15 and #20
#22 #5 or #21 Online Publication Date from Sep 2015

EMBase (Elsevier)

No.	Query
#23	#22 AND (2015:py OR 2016:py OR 2017:py OR 2018:py OR 2019:py)
#22	#5 OR #21
#21	#10 AND #11 AND #15 AND #20
#20	#16 OR #17 OR #18 OR #19
#19	'care research':ti,ab
#18	'healthcare research':ti,ab
#17	'health services evaluation':ti,ab
#16	'health services research'/exp
#15	#12 OR #13 OR #14
#14	'quality assurance':ti,ab
#13	'program evaluation':ti,ab
#12	'total quality management'/exp
#11	'health care quality'/exp
#10	#6 OR #7 OR #8 OR #9
#9	'cooperative behavior':ti,ab OR 'cooperative behaviour':ti,ab
#8	'organisational model':ti,ab OR 'organisational models':ti,ab
#7	'organizational model':ti,ab OR 'organizational models':ti,ab
#6	'organization'/exp
#5	#3 AND #4
#4	collaborative*:ti,ab
#3	#1 OR #2
#2	'quality improvement':ti,ab
#1	'quality improve':ti,ab

CINAHL (Ebsco)

#	Query
S23	S5 OR S21<br>Date: 20150101-20190315
S22	S5 OR S21
S21	S10 AND S11 AND S15 AND S20
S20	S16 OR S17 OR S18 OR S19
S19	TI Care Research OR AB Care Research
S18	TI Healthcare Research OR AB Healthcare Research
S17	TI Health Services Evaluat* OR AB Health Services Evaluat*
S16	(MH "Health Services Research+")

S15	S12 OR S13 OR S14
S14	TI Quality Assurance OR AB Quality Assurance
S13	TI Program Evaluat* OR AB Program Evaluat*
S12	TI Total Quality Management OR AB Total Quality Management
S11	(MH "Process Assessment (Health Care)+")
S10	S6 OR S7 OR S8 OR S9
S9	TI Cooperative Behav* OR AB Cooperative Behav*
S8	TI Organisational Model* OR AB Organisational Model*
S7	TI Organizational Model* OR AB Organizational Model*
S6	(MH "Organizational Change")
S5	S3 AND S4
S4	TI Collaborative* OR AB Collaborative*
S3	S1 OR S2
S2	TI Quality Improve* OR AB Quality Improve*
S1	(MH "Quality Improvement+")
