# Supplementary material for: The effectiveness of the quality improvement collaborative strategy in low- and middle-income countries: A systematic review and meta-analysis
Source: PLoS One. 2019 Oct 3;14(10):e0221919. doi: 10.1371/journal.pone.0221919 (PMC6776335; doi:10.1371/journal.pone.0221919)
Supplement: S2 File — (PDF) [file pone.0221919.s003.pdf]

Meta-analysis results, forest plots, and funnel plots

NOTE: The analysis presented here was performed with StatsDirect software (StatsDirect Ltd, England 2017) [Available from: http://www.statsdirect.com]. StatsDirect provides dropdown menus and choice buttons, instead of commands, to select the analysis method. We used the "Meta-Analysis Summary inverse variance method" to estimate the pooled effect size differences. The following results are StatsDirect output for strategy-outcome groups involving >1 study. In numerical results, commas represent decimal points (e.g., 10,3 = 10.3). The StatsDirect output provides detailed information needed to reproduce the analysis with any statistical software.

Content

1. Collaborative improvement vs. control
Outcome: 1.1 Care-seeking behavior outcomes (%). 2
Outcome: 1.2 Care-seeking behavior outcomes (continuous) 4
Outcome: 1.3 Patient behaviors not related to care-seeking (%). 7
Outcome: 1.4 Health worker practice outcomes (%). 9
Outcome: 1.5 Health worker practice outcomes (continuous) 11
Outcome: 1.6 Patient health outcomes (%). 14

2. Collaborative improvement + training vs. control
Outcome: 2.1 Patient behaviors not related to care-seeking (%). 16
Outcome: 2.2 Health worker practice outcomes (%). 18
Outcome: 2.3 Patient health outcomes (continuous) 20

3. Collaborative improvement + strengthening infrastructure + regulation and governance vs. control
Outcome: 3.1 Patient behaviors related to care-seeking (%). 22
Outcome: 3.2 Patient behaviors not related to care-seeking (%). 23

1. Collaborative improvement vs. control, outcome: 1.1 Care-seeking behavior outcomes (%).

Summary meta-analysis

Study	* Difference	SE	Approximate 95% CI		
1	10,3	6,9816	-3,383685	23,983685	Catsambas 2008-6
2	21,55	28,483387	-34,276412	77,376412	Catsambas 2008-7
3	-0,45	7,406908	-14,967272	14,067272	Catsambas 2008-9
4	3	12,759608	-22,008373	28,008373	Osibo 2017
5	28,9	20,28047	-10,848992	68,648992	Singh 2013
6	4,752	3,844866	-2,783798	12,287798	Singh 2016-1
7	5,5	4,258659	-2,846819	13,846819	Singh 2016-2
8	9,85	7,764466	-5,368074	25,068074	Singh 2016-3

Stratum	Standardized Effect	Standard Error	% Weights (fixed, random)		
1	10,3	6,9816	10,917316	10,917316	Catsambas 2008-6
2	21,55	28,483387	0,655907	0,655907	Catsambas 2008-7
3	-0,45	7,406908	9,699558	9,699558	Catsambas 2008-9
4	3	12,759608	3,26852	3,26852	Osibo 2017
5	28,9	20,28047	1,293808	1,293808	Singh 2013
6	4,752	3,844866	35,996763	35,996763	Singh 2016-1
7	5,5	4,258659	29,341343	29,341343	Singh 2016-2
8	9,85	7,764466	8,826786	8,826786	Singh 2016-3

Fixed effects (inverse variance)
Pooled * difference = 5,887928 (95% CI = 1,366652 to 10,409204)
Z (test test * Difference differs from 0) = 2,552405 P = 0,0107

Non-combinability of studies
Cochran Q = 3,12863 (df = 7) P = 0,8729
Moment-based estimate of between studies variance = 0
I_2 (inconsistency) = 0% (95% CI = 0% to 56,3%)

Random effects (DerSimonian-Laird)
Pooled * difference = 5,887928 (95% CI = 1,366652 to 10,409204)
Z (test * Difference) = 2,552405 P = 0,0107

Bias indicators
Begg-Mazumdar: Kendall's tau = 0,357143 P = 0,2751 (low power)
Egger: bias = 0,690367 (95% CI = -0,363794 to 1,744529) P = 0,1602

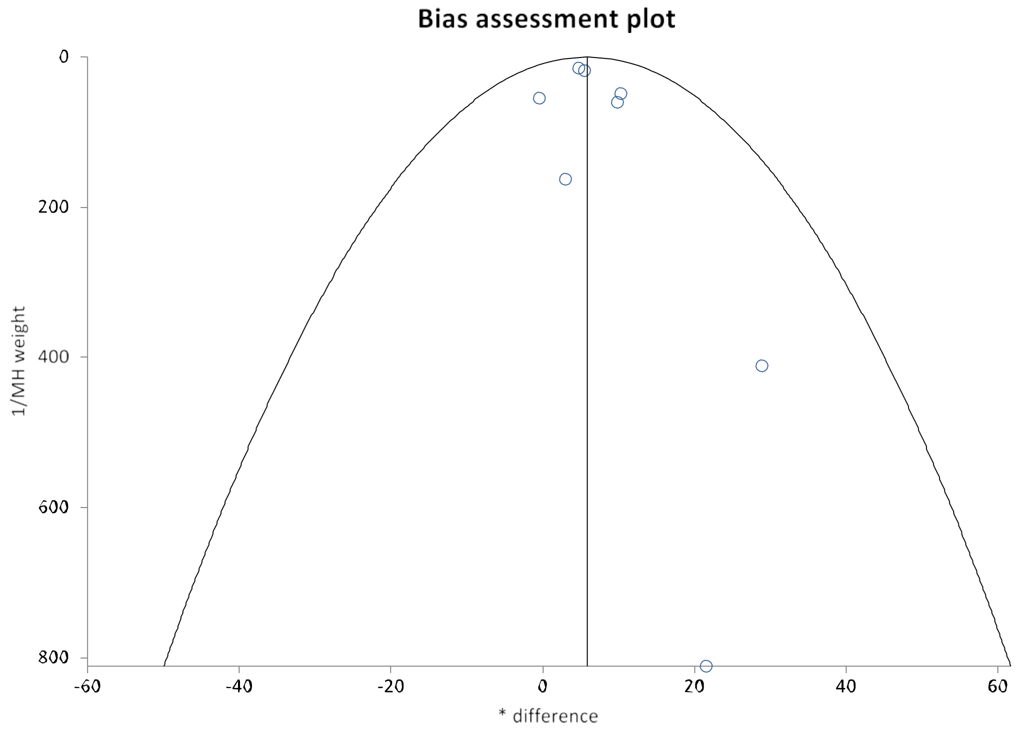

Bias assessment plot
1/MH weight
* difference
0
200
400
600
800
-60
-40
-20
0
20
40
60

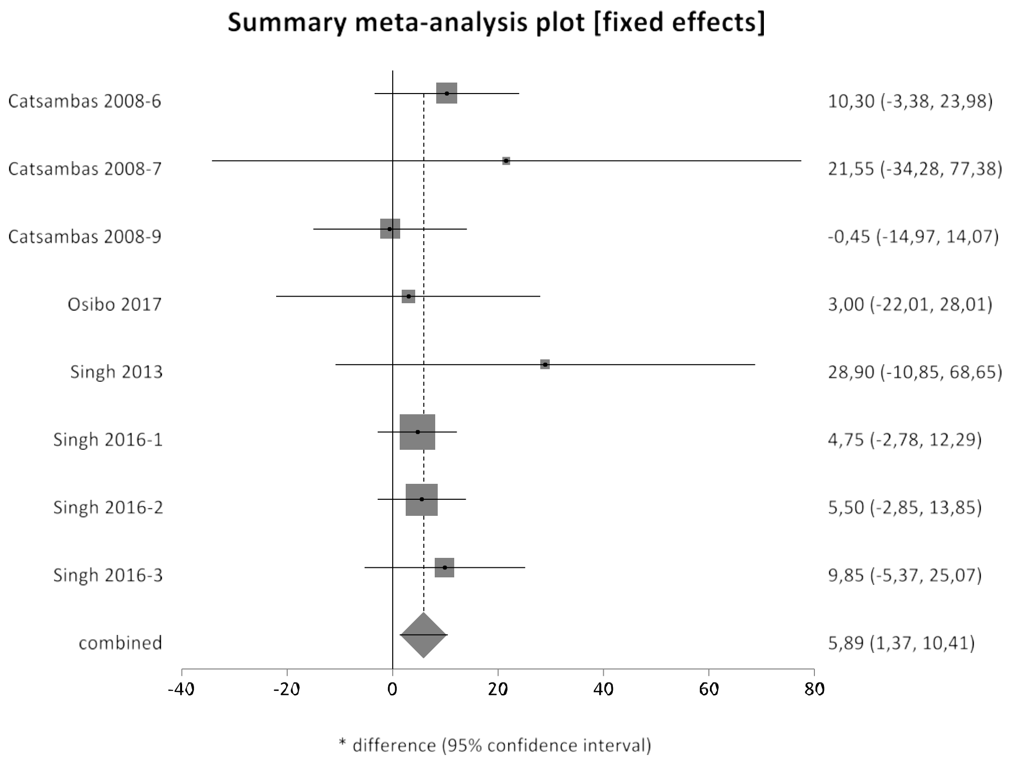

Summary meta-analysis plot [fixed effects]
Catsambas 2008-6
Catsambas 2008-7
Catsambas 2008-9
Osibo 2017
Singh 2013
Singh 2016-1
Singh 2016-2
Singh 2016-3
combined
10,30 (-3,38, 23,98)
21,55 (-34,28, 77,38)
-0,45 (-14,97, 14,07)
3,00 (-22,01, 28,01)
28,90 (-10,85, 68,65)
4,75 (-2,78, 12,29)
5,50 (-2,85, 13,85)
9,85 (-5,37, 25,07)
5,89 (1,37, 10,41)
-40
-20
0
20
40
60
80
* difference (95% confidence interval)

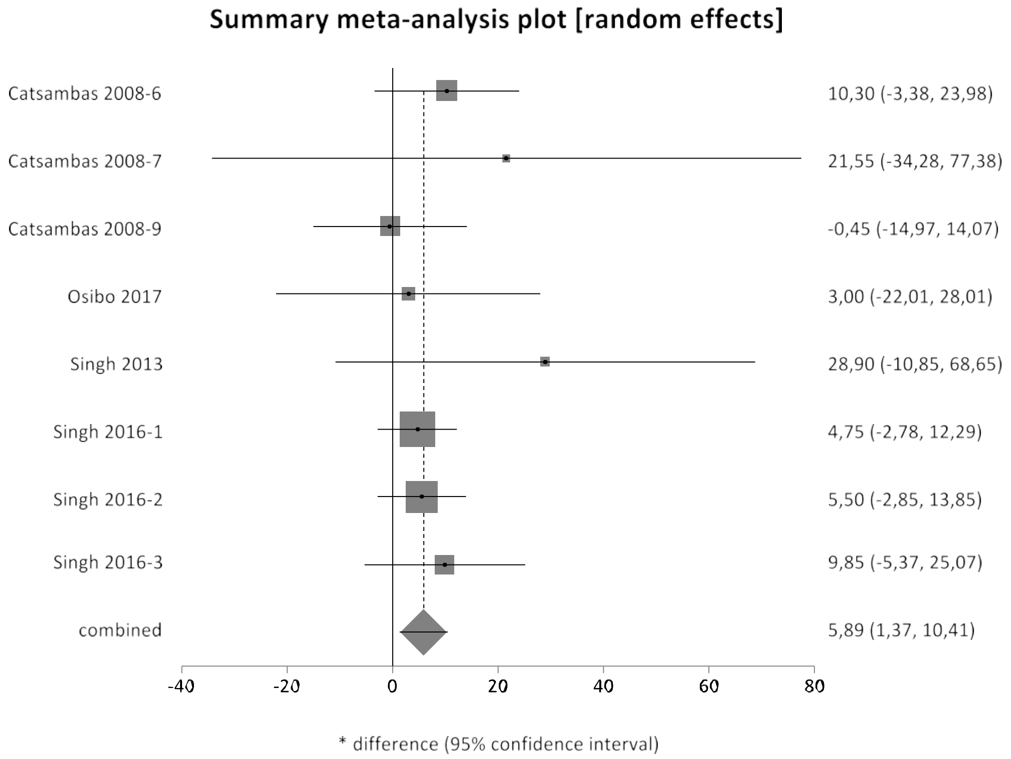

1. Collaborative improvement vs. control, outcome: 1.2 Care-seeking behavior outcomes (continuous). Summary meta-analysis

Summary meta-analysis

Study	* Difference	SE	Approximate 95% CI		
1	52,75	153,399199	-247,906906	353,406906	Crigler 2012
2	20,4	45,564999	-68,905756	109,705756	Singh 2016-1
3	71,7	60,75	-47,367812	190,767812	Singh 2016-2
4	116,1	71,291	-23,627793	255,827793	Singh 2013
5	85,7	55,435593	-22,951766	194,351766	Jaribu Pilot
6	7,1	13,43086	-19,224002	33,424002	Jaribu Imp

Stratum	Standardized Effect	Standard Error	% Weights (fixed, random)		
1	52,75	153,399199	0,619404	0,619404	Crigler 2012
2	20,4	45,564999	7,020328	7,020328	Singh 2016-1
3	71,7	60,75	3,949367	3,949367	Singh 2016-2
4	116,1	71,291	2,867812	2,867812	Singh 2013
5	85,7	55,435593	4,742886	4,742886	Jaribu Pilot
6	7,1	13,43086	80,800203	80,800203	Jaribu Imp

Fixed effects (inverse variance)
Pooled * difference = 17,721576 (95% CI = -5,940788 to 41,383941)
Z (test test * Difference differs from 0) = 1,467886 P = 0,1421

Non-combinability of studies
Cochran Q = 4,878499 (df = 5) P = 0,4309
Moment-based estimate of between studies variance = 0

I_2 (inconsistency) = 0% (95% CI = 0% to 61%)

Random effects (DerSimonian-Laird)
Pooled * difference = 17,721576 (95% CI = -5,940788 to 41,383941)
Z (test * Difference) = 1,467886 P = 0,1421

Bias indicators
Begg-Mazumdar: Kendall's tau = 0,466667 P = 0,2722 (low power)
Egger: bias = 1,125756 (95% CI = 0,078756 to 2,172756) P = 0,0405

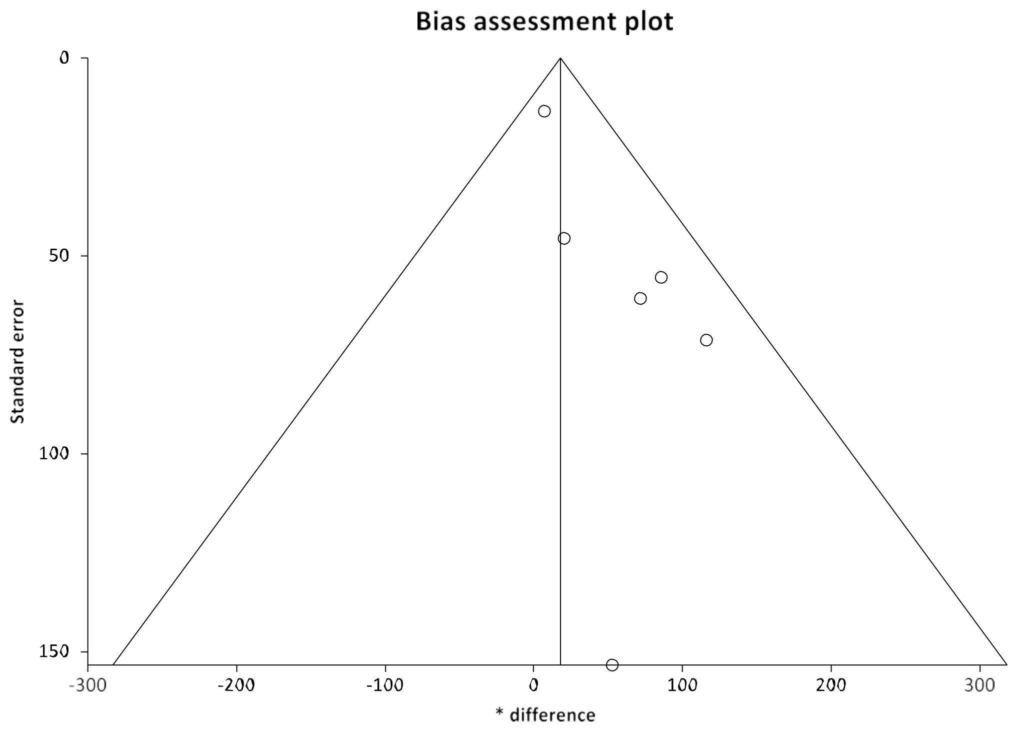

Summary meta-analysis plot [fixed effects]

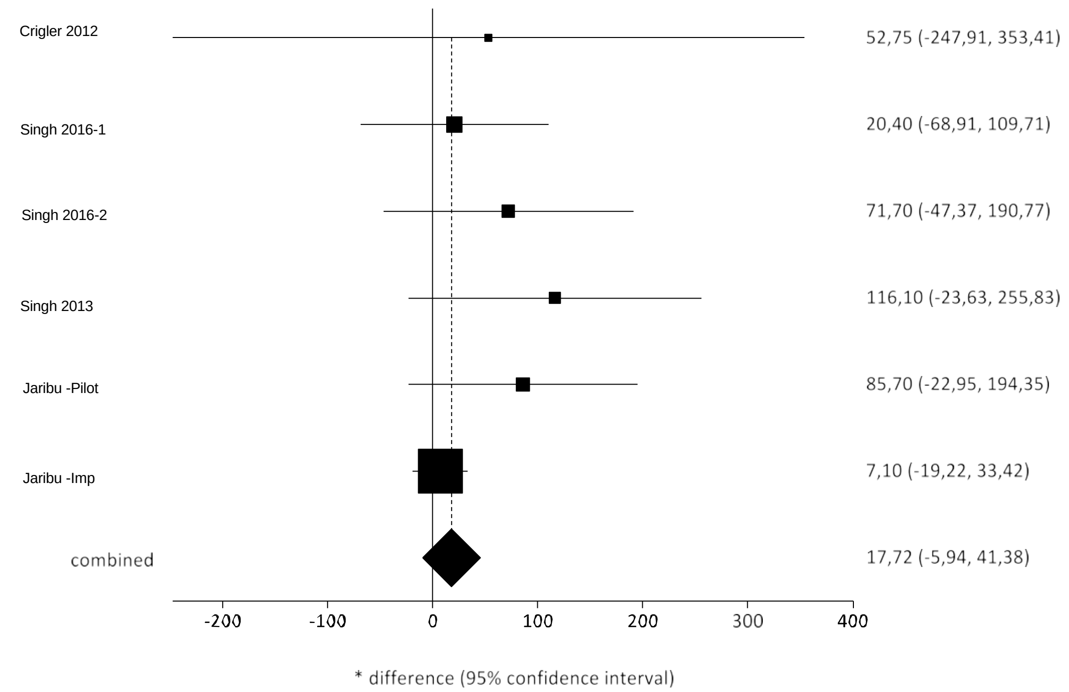

Summary meta-analysis plot [random effects]

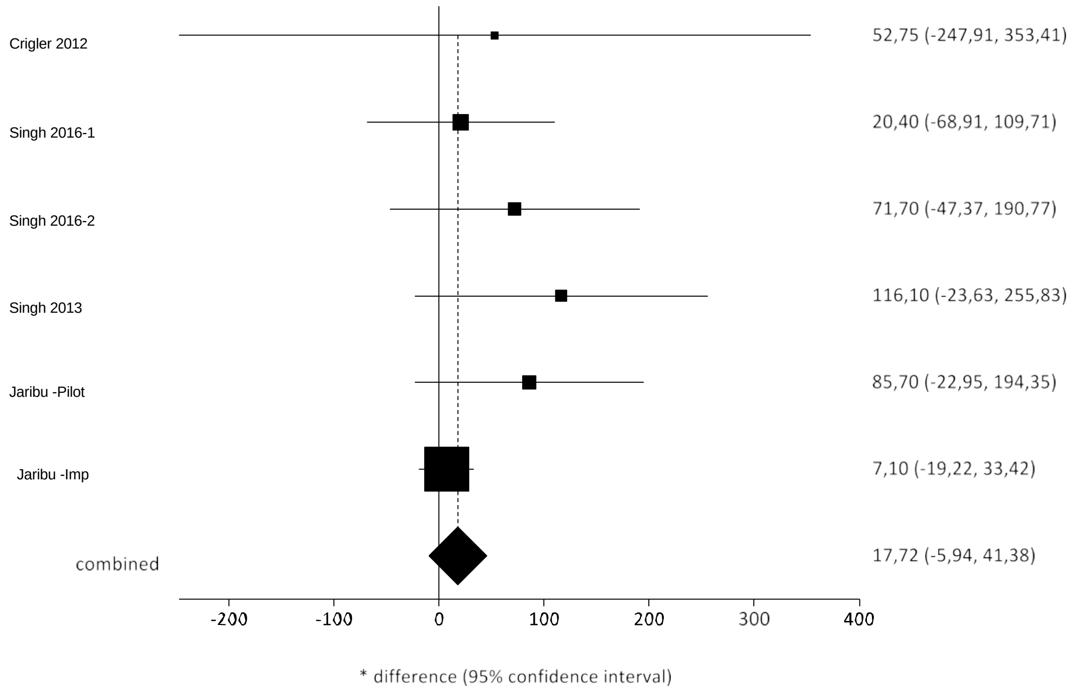

1. Collaborative improvement vs. control, outcome: 1.3 Patient behaviors not related to care-seeking (%).
Summary meta-analysis

Study	* Difference	SE	Approximate 95% CI		
1	26,2	7,4982	11,503798	40,896202	Catsambas 2008-10
2	17,6	3,5744	10,594305	24,605695	Catsambas 2008-11
3	12,5	1,1266	10,291905	14,708095	Catsambas 2008-6

Stratum	Standardized Effect	Standard Error	% Weights (fixed, random)		
1	26,2	7,4982	2,01217	13,263826	Catsambas 2008-10
2	17,6	3,5744	8,854672	32,835536	Catsambas 2008-11
3	12,5	1,1266	89,133158	53,900637	Catsambas 2008-6

Fixed effects (inverse variance)
Pooled * difference = 13,227256 (95% CI = 11,142585 to 15,311926)
Z (test test * Difference differs from 0) = 12,43599 P < 0,0001

Non-combinability of studies
Cochran Q = 4,906594 (df = 2) P = 0,086
Moment-based estimate of between studies variance = 16,667646
I_2 (inconsistency) = 59,2% (95% CI = 0% to 86,6%)

Random effects (DerSimonian-Laird)
Pooled * difference = 15,991757 (95% CI = 9,897533 to 22,085981)
Z (test * Difference) = 5,14311 P < 0,0001

Bias indicators
Begg-Mazumdar: Kendall's <too few strata> *
Egger: bias = <too few strata> (95% CI = * to *) P = *

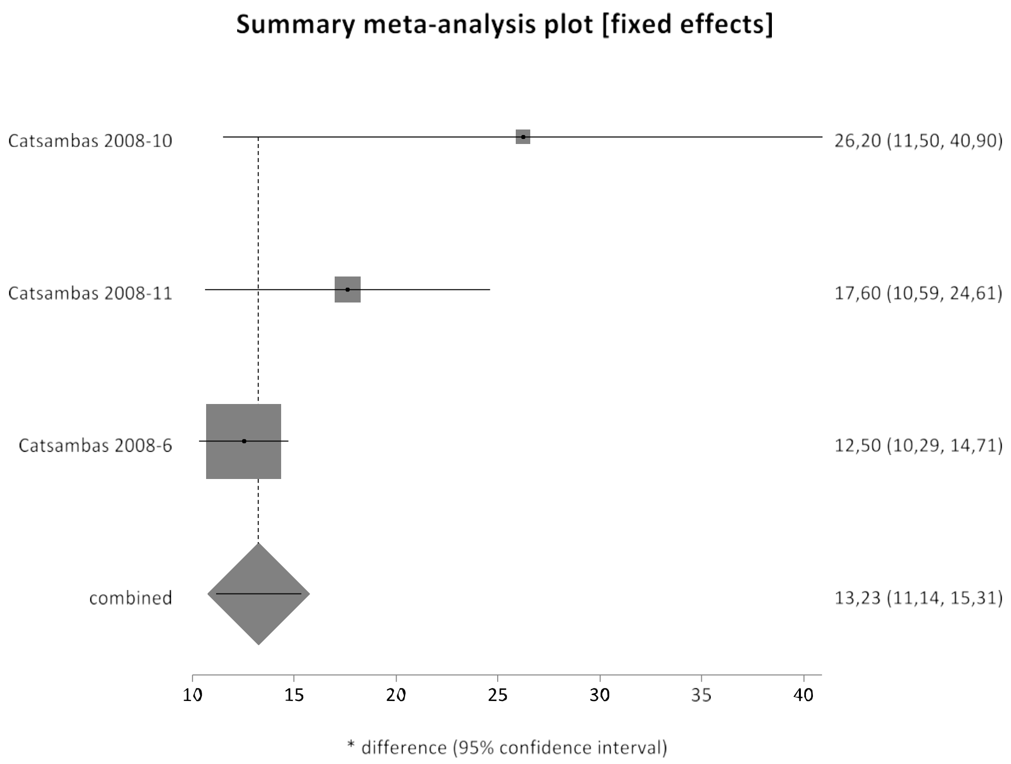
Summary meta-analysis plot [fixed effects]
Catsambas 2008-10
26,20 (11,50, 40,90)
Catsambas 2008-11
17,60 (10,59, 24,61)
Catsambas 2008-6
12,50 (10,29, 14,71)
combined
13,23 (11,14, 15,31)
10
15
20
25
30
35
40
* difference (95% confidence interval)

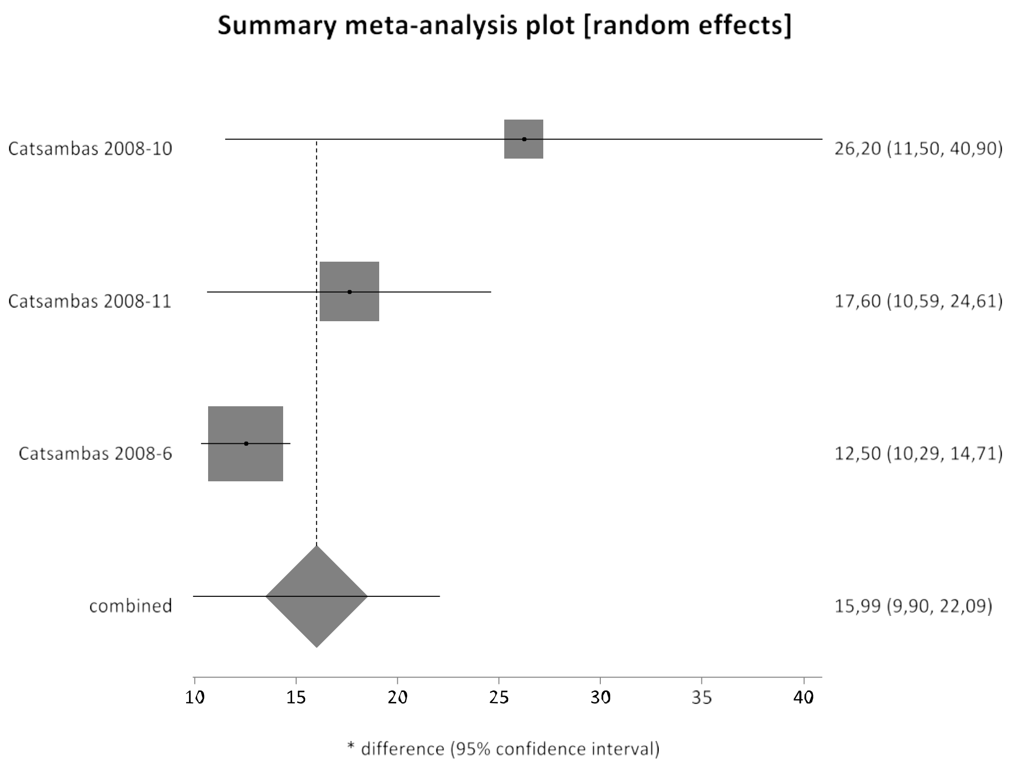
Summary meta-analysis plot [random effects]
Catsambas 2008-10
26,20 (11,50, 40,90)
Catsambas 2008-11
17,60 (10,59, 24,61)
Catsambas 2008-6
12,50 (10,29, 14,71)
combined
15,99 (9,90, 22,09)
10
15
20
25
30
35
40
* difference (95% confidence interval)

1. Collaborative improvement vs. control, outcome: 1.4 Health worker practice outcomes (%).
Summary meta-analysis

Study	* Difference	SE	Approximate 95% CI		
1	71,15	12,23982	47,160394	95,139606	Barceló 2010
2	19,6	13,751256	-7,351966	46,551966	Catsambas 2008-10
3	14,3	12,46081	-10,122739	38,722739	Catsambas 2008-12
4	34,3	9,9103	14,876169	53,723831	Catsambas 2008-4
5	49,7	26,40644	-2,055671	101,455671	Catsambas 2008-5
6	27,2	15,635106	-3,444244	57,844244	Catsambas 2008-9
7	64	12,189755	40,108519	87,891481	Chitashvili 2017
8	8,1	24,1	-39,135133	55,335133	Crigler 2012
9	30,15	7,807667	14,847254	45,452746	N'Guessan 2011

Stratum	Standardized Effect	Standard Error	% Weights (fixed, random)		
1	71,15	12,23982	11,320012	12,427115	Barceló 2010
2	19,6	13,751256	8,968343	11,320192	Catsambas 2008-10
3	14,3	12,46081	10,922056	12,260529	Catsambas 2008-12
4	34,3	9,9103	17,267257	14,258434	Catsambas 2008-4
5	49,7	26,40644	2,432077	5,259548	Catsambas 2008-5
6	27,2	15,635106	6,937377	10,057712	Catsambas 2008-9
7	64	12,189755	11,413188	12,465063	Chitashvili 2017
8	8,1	24,1	2,919866	5,995292	Crigler 2012
9	30,15	7,807667	27,819825	15,956113	N'Guessan 2011

Fixed effects (inverse variance)
Pooled * difference = 36,320842 (95% CI = 28,249485 to 44,392199)
Z (test test * Difference differs from 0) = 8,819774 P < 0,0001

Non-combinability of studies
Cochran Q = 20,489284 (df = 8) P = 0,0086
Moment-based estimate of between studies variance = 251,931713
I_2 (inconsistency) = 61% (95% CI = 0% to 79,4%)

Random effects (DerSimonian-Laird)
Pooled * difference = 36,328269 (95% CI = 22,479587 to 50,176951)
Z (test * Difference) = 5,141435 P < 0,0001

Bias indicators
Begg-Mazumdar: Kendall's tau = 0 P = 0,9195 (low power)
Egger: bias = -0,114721 (95% CI = -4,266469 to 4,037028) P = 0,9497

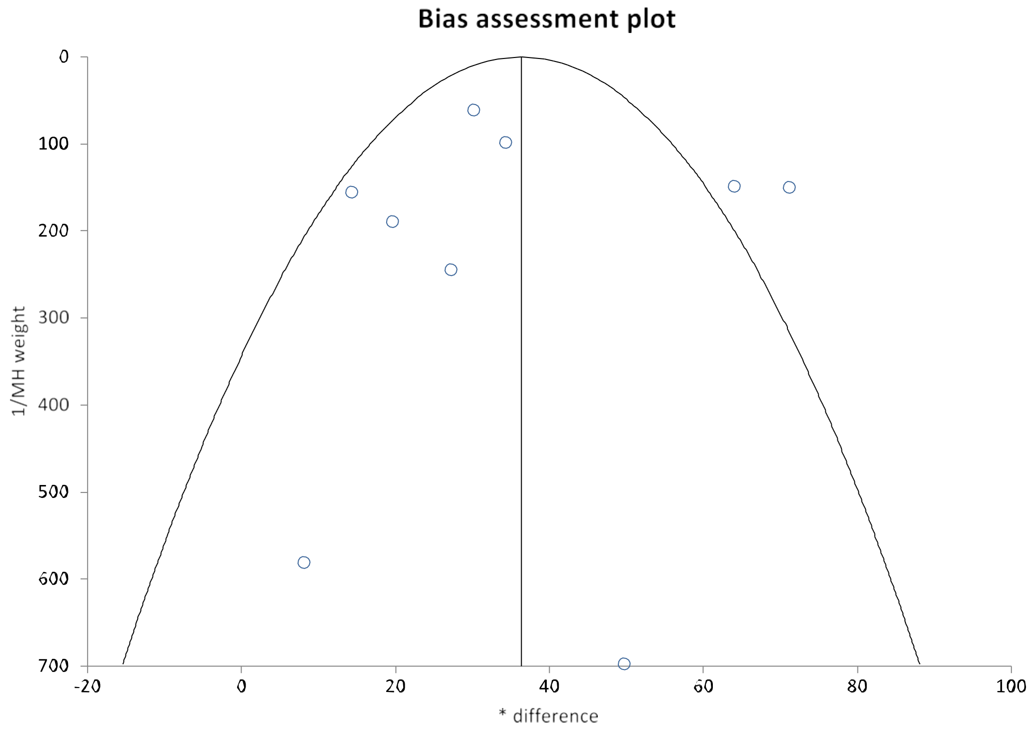

Bias assessment plot
1/MH weight
0
100
200
300
400
500
600
700
-20
0
20
40
60
80
100
* difference

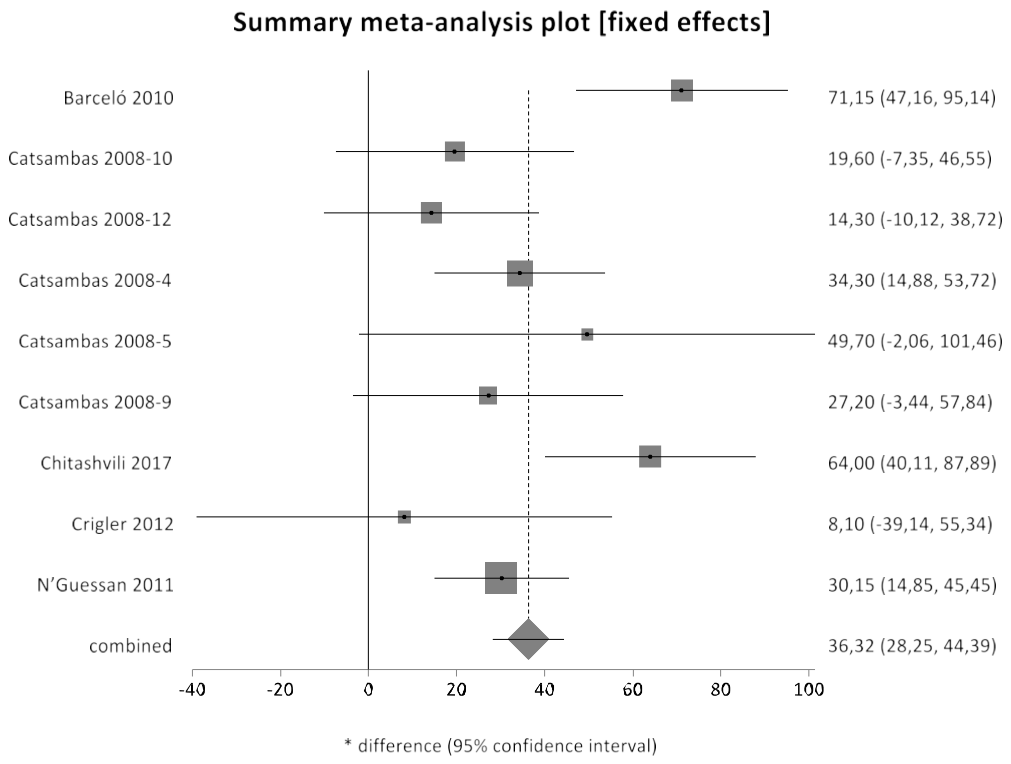

Summary meta-analysis plot [fixed effects]
Barceló 2010
Catsambas 2008-10
Catsambas 2008-12
Catsambas 2008-4
Catsambas 2008-5
Catsambas 2008-9
Chitashvili 2017
Crigler 2012
N'Guessan 2011
combined
71,15 (47,16, 95,14)
19,60 (-7,35, 46,55)
14,30 (-10,12, 38,72)
34,30 (14,88, 53,72)
49,70 (-2,06, 101,46)
27,20 (-3,44, 57,84)
64,00 (40,11, 87,89)
8,10 (-39,14, 55,34)
30,15 (14,85, 45,45)
36,32 (28,25, 44,39)
-40
-20
0
20
40
60
80
100
* difference (95% confidence interval)

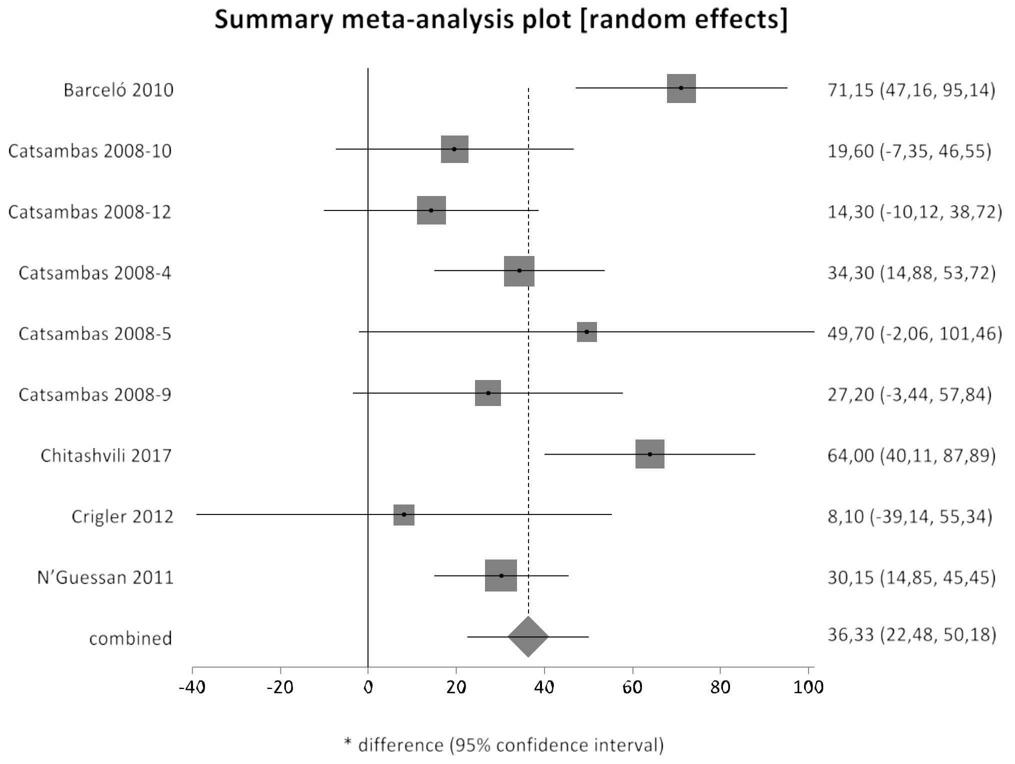

1. Collaborative improvement vs. control, outcome: 1.5 Health worker practice outcomes (continuous)

Summary meta-analysis

Study	* Difference	SE	Approximate 95% CI		
1	4,3	4,058258	-3,654039	12,254039	Casambas 2008-5
2	-28,2	45,0155	-116,428759	60,028759	Casambas 2008-6
3	135,2	120,381639	-100,743676	371,143676	Jaribu 2018- Imp

Stratum	Standardized Effect	Standard Error	% Weights (fixed, random)		
1	4,3	4,058258	99,08211	99,08211	Casambas 2008-5
2	-28,2	45,0155	0,805286	0,805286	Casambas 2008-6
3	135,2	120,381639	0,112604	0,112604	Jaribu 2018- Imp

Fixed effects (inverse variance)
Pooled * difference = 4,185681 (95% CI = -3,73177 to 12,103131)
Z (test test * Difference differs from 0) = 1,036165 P = 0,3001

Non-combinability of studies
Cochran Q = 1,70283 (df = 2) P = 0,4268
Moment-based estimate of between studies variance = 0
I_2 (inconsistency) = 0% (95% CI = 0% to 72,9%)

Random effects (DerSimonian-Laird)
Pooled * difference = 4,185681 (95% CI = -3,73177 to 12,103131)
Z (test * Difference) = 1,036165 P = 0,3001

Bias indicators
Begg-Mazumdar: Kendall's <too few strata> *
Egger: bias = <too few strata> (95% CI = * to *) P = *

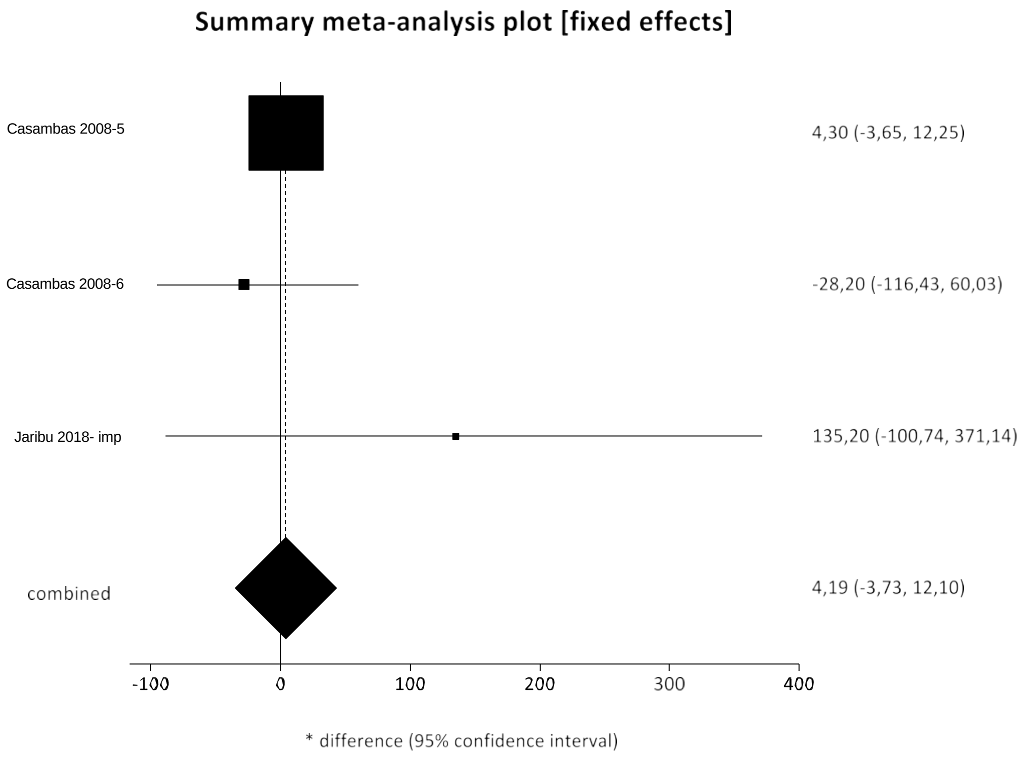

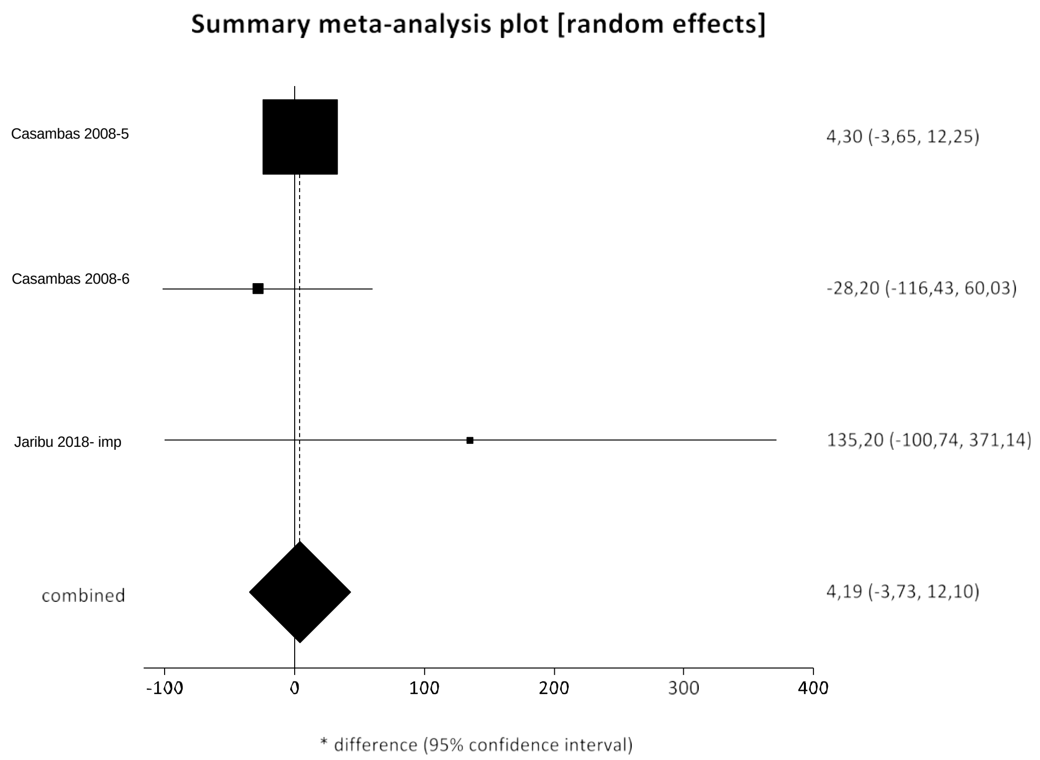
Summary meta-analysis plot [random effects]
Casambas 2008-5
4,30 (-3,65, 12,25)
Casambas 2008-6
-28,20 (-116,43, 60,03)
Jaribu 2018- imp
135,20 (-100,74, 371,14)
combined
4,19 (-3,73, 12,10)
-100
0
100
200
300
400
* difference (95% confidence interval)

1. Collaborative improvement vs. control, outcome: 1.6 Patient health outcomes (%).
Summary meta-analysis

Study	* Difference	SE	Approximate 95% CI		
1	11,4	13,636159	-15,32638	38,12638	Barceló 2010
2	0,3	8,4695	-16,299915	16,899915	Catsambas 2008-11
3	-15	9,8196	-34,246062	4,246062	Catsambas 2008-6

Stratum	Standardized Effect	Standard Error	% Weights (fixed, random)		
1	11,4	13,636159	18,113981	21,438082	Barceló 2010
2	0,3	8,4695	46,955067	43,158366	Catsambas 2008-11
3	-15	9,8196	34,930952	35,403551	Catsambas 2008-6

Fixed effects (inverse variance)
Pooled * difference = -3,033784 (95% CI = -14,408671 to 8,341103)
Z (test test * Difference differs from 0) = -0,52274 P = 0,6012

Non-combinability of studies
Cochran Q = 2,760346 (df = 2) P = 0,2515
Moment-based estimate of between studies variance = 40,996056
I_2 (inconsistency) = 27,5% (95% CI = 0% to 79,6%)

Random effects (DerSimonian-Laird)
Pooled * difference = -2,737116 (95% CI = -16,408026 to 10,933794)
Z (test * Difference) = -0,392413 P = 0,6948

Bias indicators
Begg-Mazumdar: Kendall's <too few strata> *
Egger: bias = <too few strata> (95% CI = * to *) P = *

Summary meta-analysis plot [fixed effects]

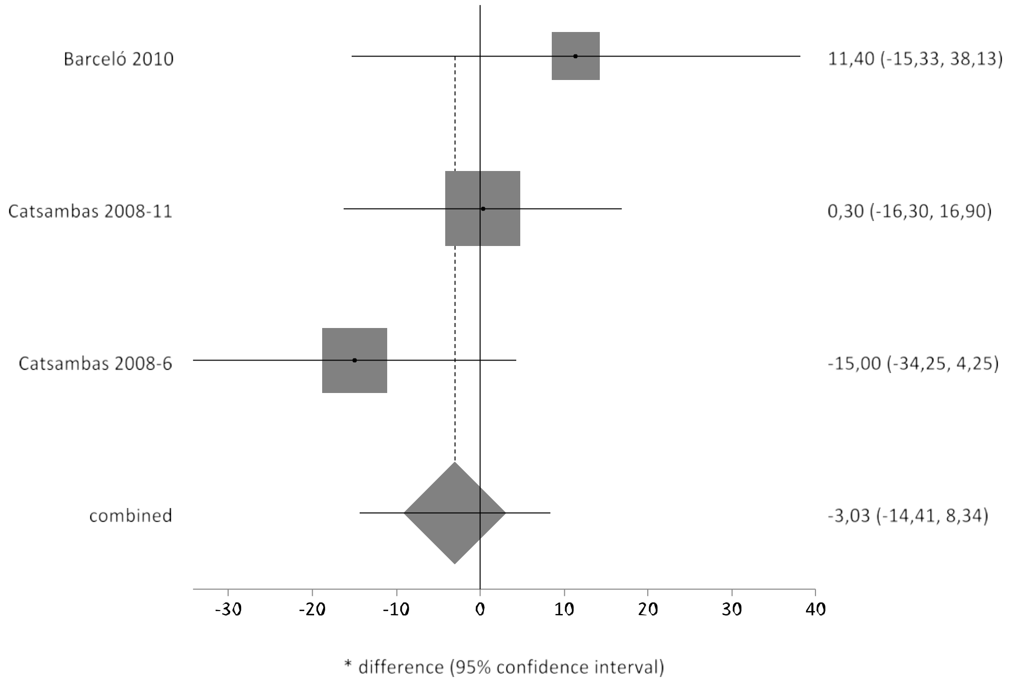

Summary meta-analysis plot [random effects]

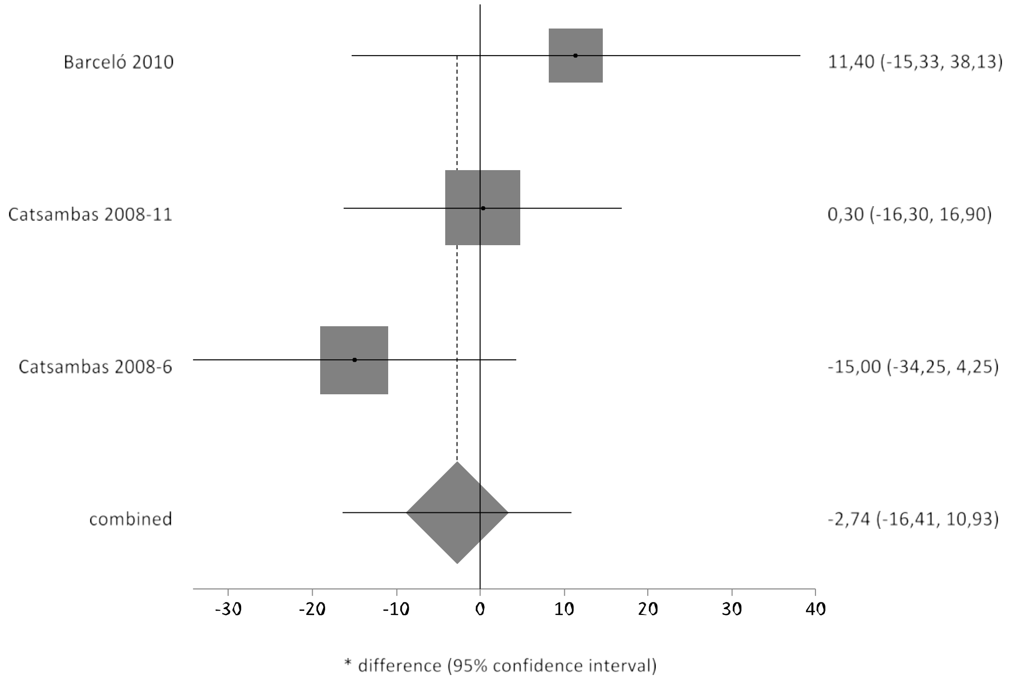

2. Collaborative improvement + training vs. control, outcome: 2.1 Patient behaviors not related to care-seeking (%). Summary meta-analysis

Study	* Difference	SE	Approximate 95% CI		
1	78,7	3,8606	71,133363	86,266637	Catsambas 2008-1
2	96,7	2,199	92,390039	101,009961	Catsambas 2008-2

Stratum	Standardized Effect	Standard Error	% Weights (fixed, random)		
1	78,7	3,8606	24,496666	48,446199	Catsambas 2008-1
2	96,7	2,199	75,503334	51,553801	Catsambas 2008-2

Fixed effects (inverse variance)
Pooled * difference = 92,2906 (95% CI = 88,545561 to 96,03564)
Z (test test * Difference differs from 0) = 48,300227 P < 0,0001

Non-combinability of studies
Cochran Q = 16,413512 (df = 1) P < 0,0001
Moment-based estimate of between studies variance = 152,130083
I_2 (inconsistency) = 93,9% (95% CI = *% to *%)

Random effects (DerSimonian-Laird)
Pooled * difference = 87,979684 (95% CI = 70,348528 to 105,610841)
Z (test * Difference) = 9,780244 P < 0,0001

Bias indicators
Begg-Mazumdar: Kendall's <too few strata> *
Egger: bias = <too few strata> (95% CI = * to *) P = *

Summary meta-analysis plot [fixed effects]

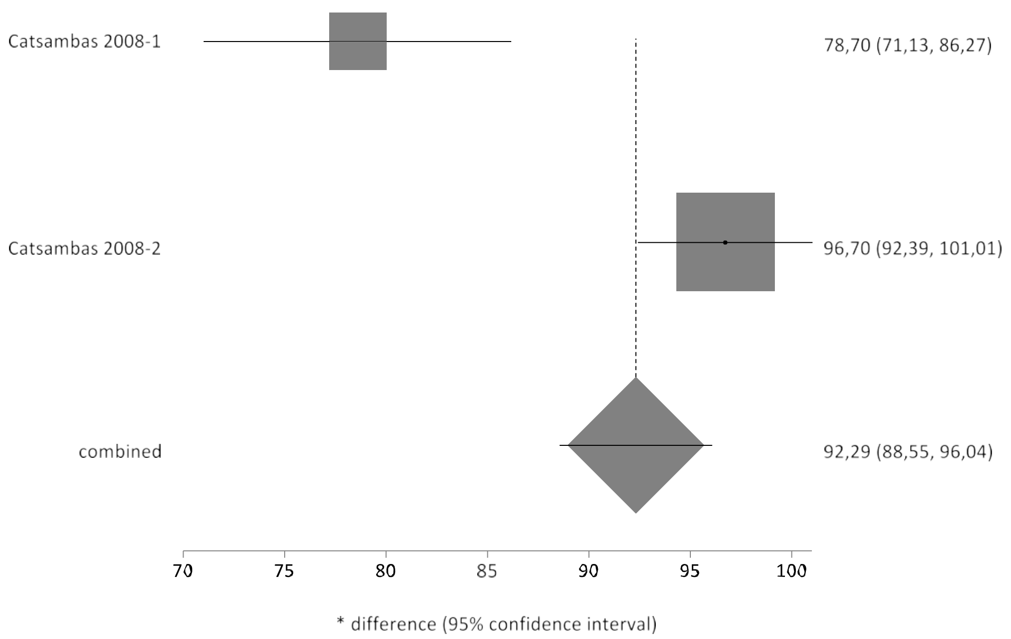

Summary meta-analysis plot [random effects]

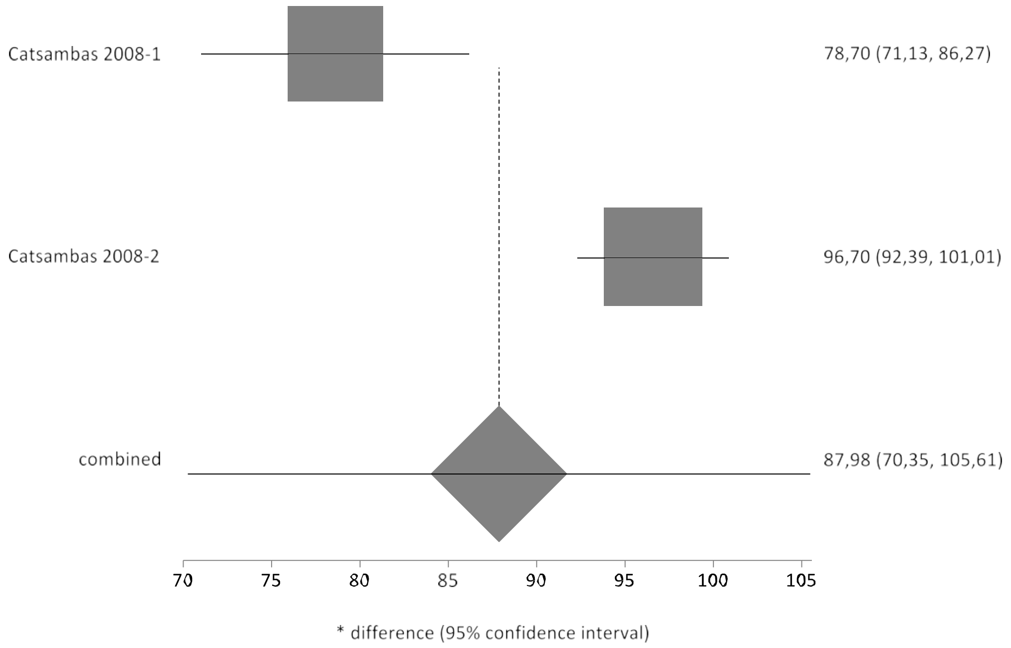

2. Collaborative improvement + training vs. control, outcome: 2.2 Health worker practice outcomes (%). Summary meta-analysis

Study	* Difference	SE	Approximate 95% CI		
1	65,95	3,629204	58,836892	73,063108	Catsambas 2008-1
2	89,6	16,207633	57,833622	121,366378	Catsambas 2008-2
3	35,3	10,28542	15,140947	55,459053	Catsambas 2008-3
4	60,85	4,503655	52,022999	69,677001	Westercamp 2017

Stratum	Standardized Effect	Standard Error	% Weights (fixed, random)		
1	65,95	3,629204	54,824174	35,703222	Catsambas 2008-1
2	89,6	16,207633	2,748878	11,111408	Catsambas 2008-2
3	35,3	10,28542	6,825755	19,600716	Catsambas 2008-3
4	60,85	4,503655	35,601193	33,584654	Westercamp 2017

Fixed effects (inverse variance)
Pooled * difference = 62,692355 (95% CI = 57,425572 to 67,959138)
Z (test test * Difference differs from 0) = 23,330133 P < 0,0001

Non-combinability of studies
Cochran Q = 10,822027 (df = 3) P = 0,0127
Moment-based estimate of between studies variance = 99,568713
I_2 (inconsistency) = 72,3% (95% CI = 0% to 88,1%)

Random effects (DerSimonian-Laird)
Pooled * difference = 60,857411 (95% CI = 48,422563 to 73,292259)
Z (test * Difference) = 9,592263 P < 0,0001

Bias indicators
Begg-Mazumdar: Kendall's tau = 0 P = 0,75 (low power)
Egger: bias = -0,57956 (95% CI = -11,054836 to 9,895717) P = 0,834

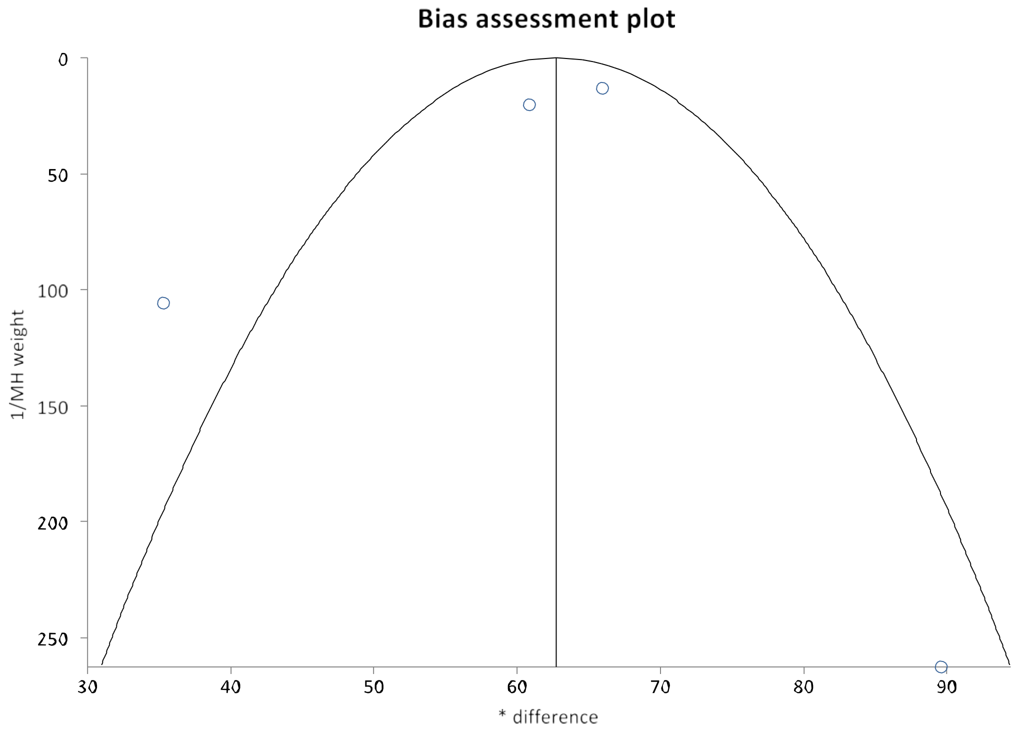
Bias assessment plot
0
50
100
150
200
250
1/MH weight
30
40
50
60
70
80
90
* difference

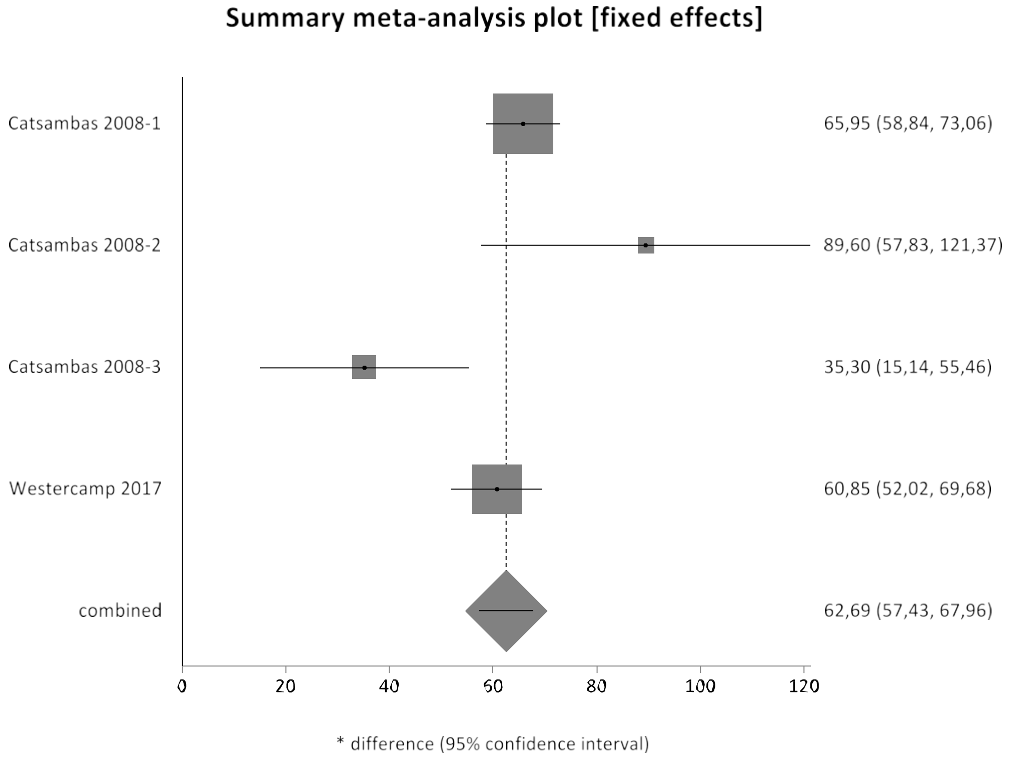
Summary meta-analysis plot [fixed effects]
Catsambas 2008-1
65,95 (58,84, 73,06)
Catsambas 2008-2
89,60 (57,83, 121,37)
Catsambas 2008-3
35,30 (15,14, 55,46)
Westercamp 2017
60,85 (52,02, 69,68)
combined
62,69 (57,43, 67,96)
0
20
40
60
80
100
120
* difference (95% confidence interval)

Summary meta-analysis plot [random effects]

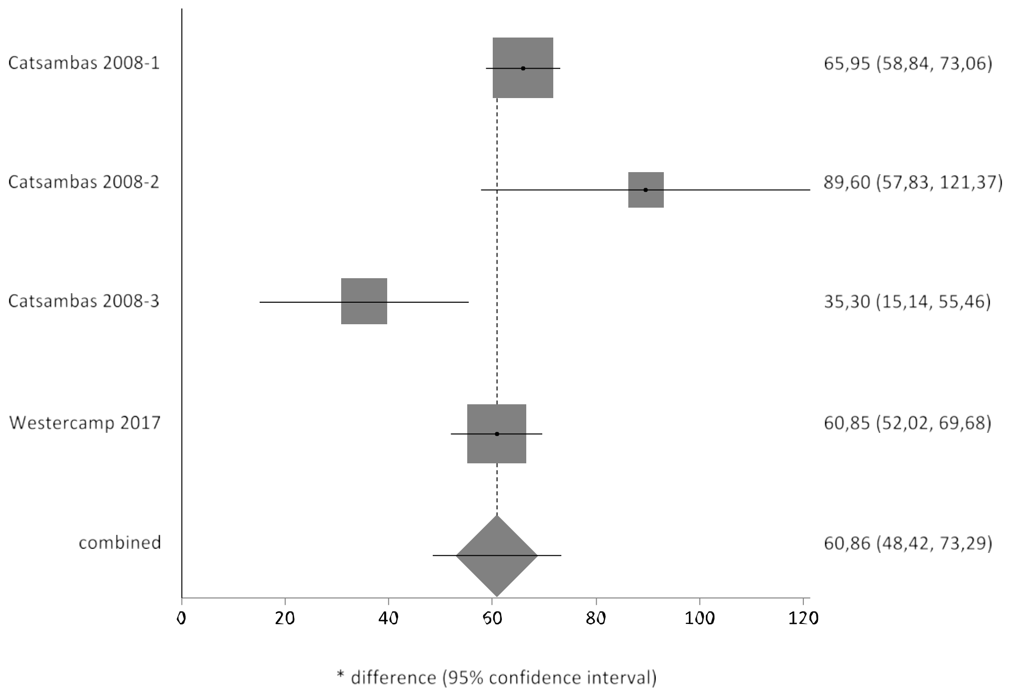

2. Collaborative improvement + training vs. control, outcome: 2.3 Patient health outcomes (continuous)

Summary meta-analysis

Study	* Difference	SE	Approximate 95% CI		
1	96	23,041074	50,840324	141,159676	Catsambas 2008-1
2	127,1	194,274725	-253,671464	507,871464	Catsambas 2008-2

Stratum	Standardized Effect	Standard Error	% Weights (fixed, random)		
1	96	23,041074	98,612904	98,612904	Catsambas 2008-1
2	127,1	194,274725	1,387096	1,387096	Catsambas 2008-2

Fixed effects (inverse variance)
Pooled * difference = 96,431387 (95% CI = 51,586009 to 141,276765)
Z (test test * Difference differs from 0) = 4,214527 P < 0,0001

Non-combinability of studies
Cochran Q = 0,025271 (df = 1) P = 0,8737
Moment-based estimate of between studies variance = 0
I_2 (inconsistency) = 0% (95% CI = *% to *%)

Random effects (DerSimonian-Laird)

Pooled * difference = 96,431387 (95% CI = 51,586009 to 141,276765)
Z (test * Difference) = 4,214527 P < 0,0001

<u>Bias indicators</u>
Begg-Mazumdar: Kendall's <too few strata> *
Egger: bias = <too few strata> (95% CI = * to *) P = *

Summary meta-analysis plot [fixed effects]

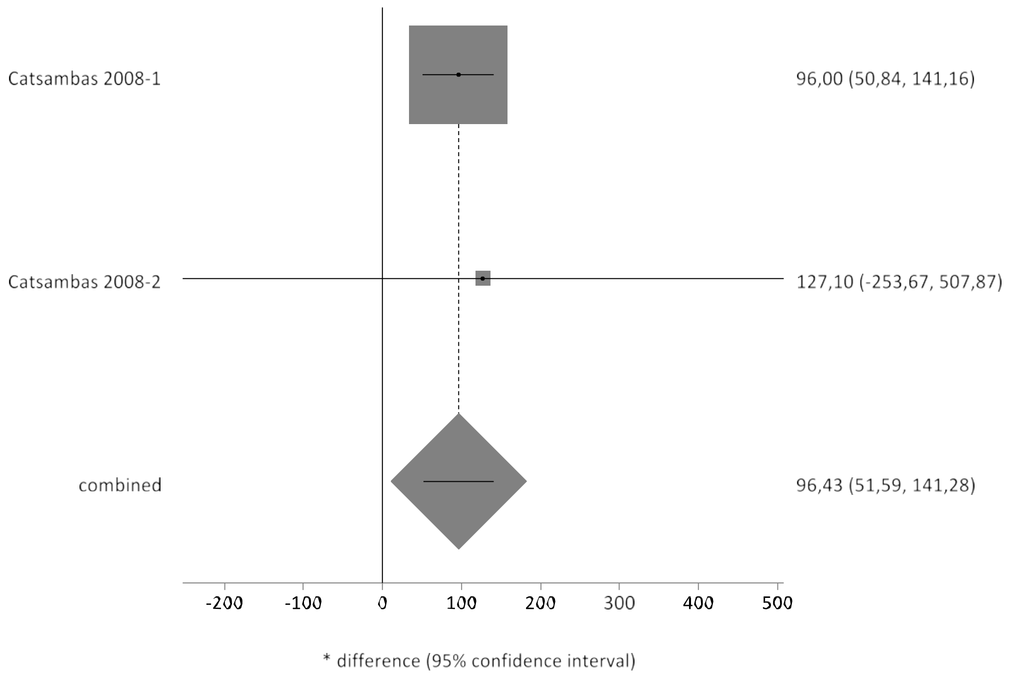

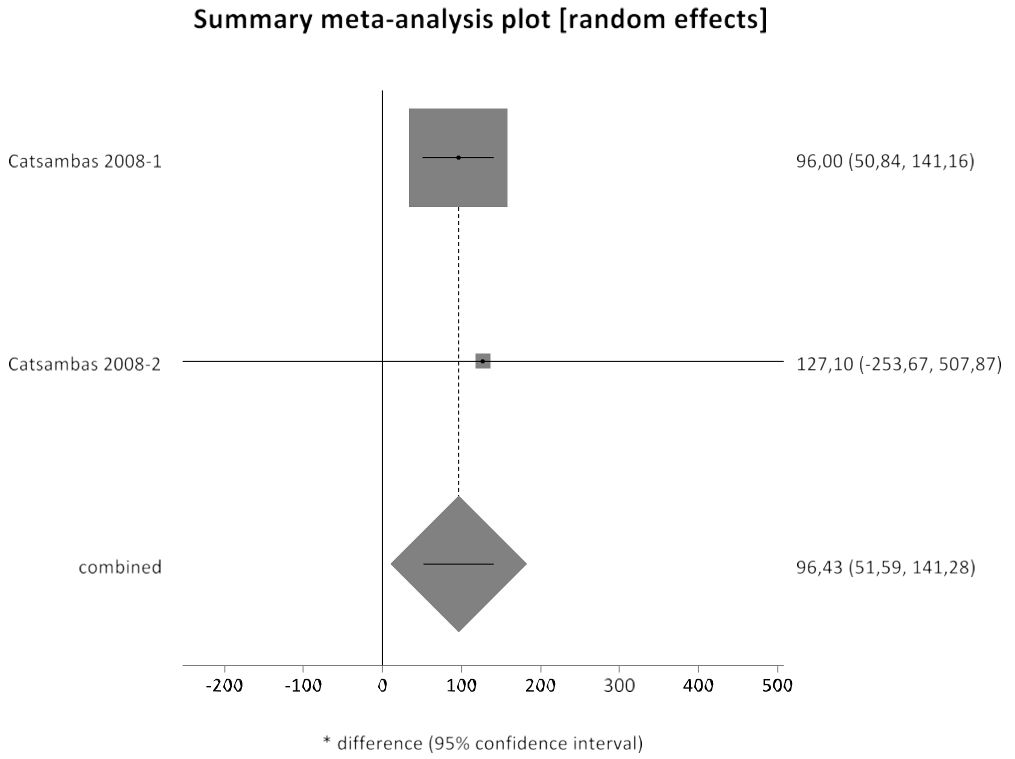

3. Collaborative improvement + strengthening infrastructure + regulation and governance vs. control, outcome: 3.1 Patient behaviors related to care-seeking (%).

Summary meta-analysis

Study	* Difference	SE	Approximate 95% CI		
1	16,5	4,47742	7,724419	25,275581	Waiswa 2017-1
2	2,5	3,855342	-5,056331	10,056331	Waiswa 2017-2

Stratum	Standardized Effect	Standard Error	% Weights (fixed, random)		
1	16,5	4,47742	42,575928	48,677646	Waiswa 2017-1
2	2,5	3,855342	57,424072	51,322354	Waiswa 2017-2

Fixed effects (inverse variance)
Pooled * difference = 8,46063 (95% CI = 2,734543 to 14,186717)
Z (test test * Difference differs from 0) = 2,895962 P = 0,0038

Non-combinability of studies
Cochran Q = 5,614285 (df = 1) P = 0,0178
Moment-based estimate of between studies variance = 80,544526
I_2 (inconsistency) = 82,2% (95% CI = *% to *%)

Random effects (DerSimonian-Laird)
Pooled * difference = 9,31487 (95% CI = -4,400078 to 23,029819)
Z (test * Difference) = 1,331161 P = 0,1831

Bias indicators
Begg-Mazumdar: Kendall's <too few strata> *
Egger: bias = <too few strata> (95% CI = * to *) P = *

Summary meta-analysis plot [fixed effects]

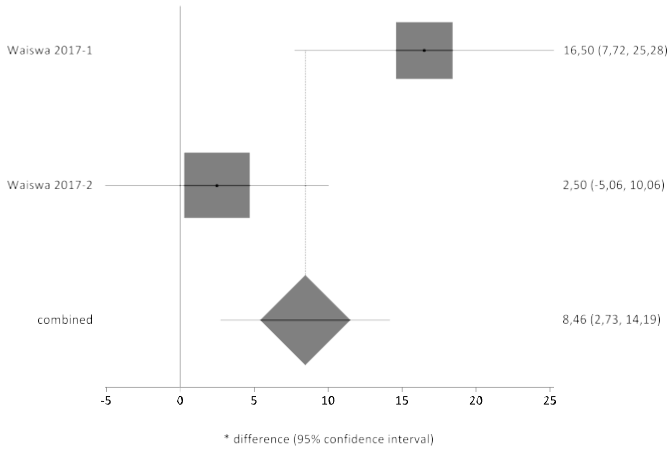

Summary meta-analysis plot [random effects]

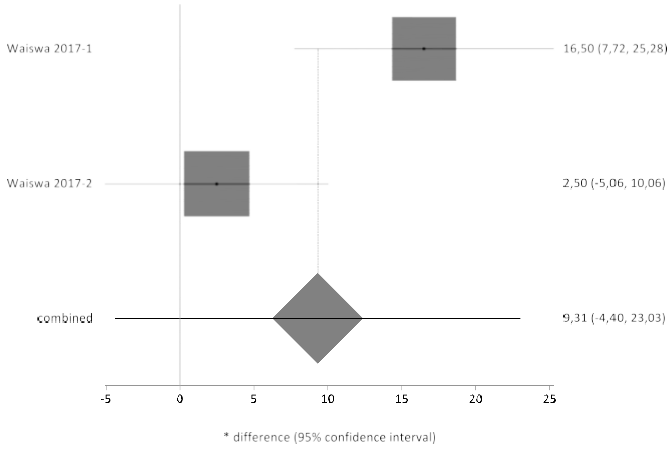

3. Collaborative improvement + strengthening infrastructure + regulation & governance vs. control, outcome: 3.2 Patient behaviors not related to care-seeking (%).
Summary meta-analysis

Study	* Difference	SE	Approximate 95% CI		
1	-1,5	6,42652	-14,095748	11,095748	Waiswa 2017-1
2	-4	5,329165	-14,444971	6,444971	Waiswa 2017-2

Stratum	Standardized Effect	Standard Error	% Weights (fixed, random)

1	-1,5	6,42652	40,745958	40,745958	Waiswa 2017-1
2	-4	5,329165	59,254042	59,254042	Waiswa 2017-2

Fixed effects (inverse variance)
Pooled * difference = -2,981351 (95% CI = -11,02154 to 5,058838)
Z (test test * Difference differs from 0) = -0,726767 P = 0,4674

Non-combinability of studies
Cochran Q = 0,08967 (df = 1) P = 0,7646
Moment-based estimate of between studies variance = 0
I_2 (inconsistency) = 0% (95% CI = *% to *%)

Random effects (DerSimonian-Laird)
Pooled * difference = -2,981351 (95% CI = -11,02154 to 5,058838)
Z (test * Difference) = -0,726767 P = 0,4674

Bias indicators
Begg-Mazumdar: Kendall's <too few strata> *
Egger: bias = <too few strata> (95% CI = * to *) P = *

Summary meta-analysis plot [fixed effects]

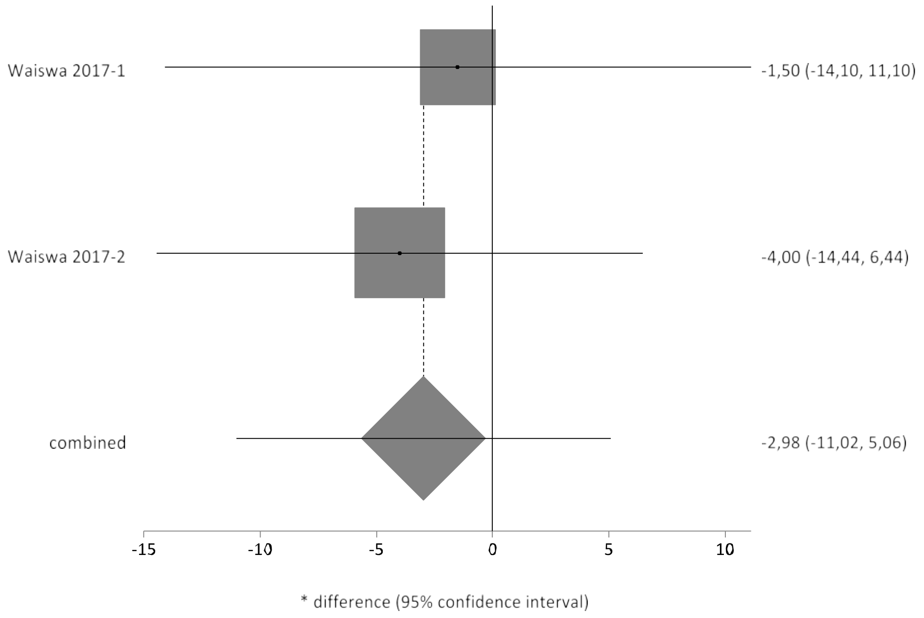

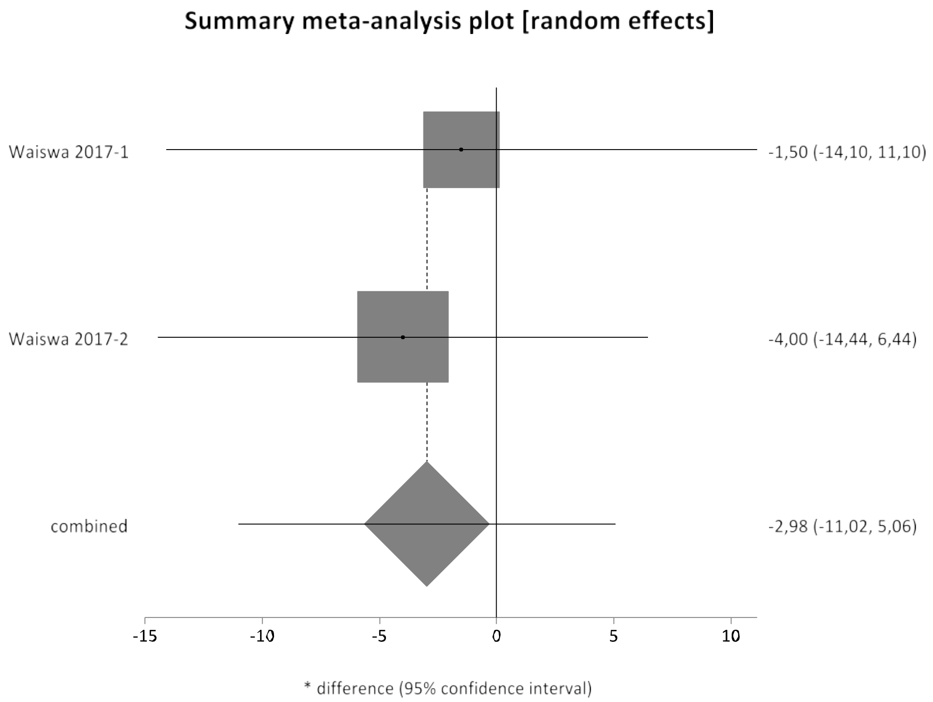
Summary meta-analysis plot [random effects]
Waiswa 2017-1
-1,50 (-14,10, 11,10)
Waiswa 2017-2
-4,00 (-14,44, 6,44)
combined
-2,98 (-11,02, 5,06)
-15
-10
-5
0
5
10
* difference (95% confidence interval)
