# Supplementary material for: The effectiveness of the quality improvement collaborative strategy in low- and middle-income countries: A systematic review and meta-analysis
Source: PLoS One. 2019 Oct 3;14(10):e0221919. doi: 10.1371/journal.pone.0221919 (PMC6776335; doi:10.1371/journal.pone.0221919)
Supplement: S3 File — (PDF) [file pone.0221919.s004.pdf]

Sensitivity analysis and list of excluded studies

Sensitivity analysis: random effects meta-analysis considering all effect sizes individually

Strategy	Outcomes	Type of Outcome*	No. of studies / comparisons (No. of effect sizes)	Mean effect size from random-effects meta-analysis (95% CI; I²)
**QIC: improvement only** (HF-based HCPs; median follow-up 11.5 months)	**Patient health outcomes**	%	3 / 3 (5)	**-1.6** (-12.5 to 9.2; 0%)
		Cont.	3 / 3 (6)	**non-evaluable****
	**Patient behaviors related to care-seeking**	%	8 / 8 (15)	**6.2** (1.4 to 11.0; 44%)
		Cont.	6 / 6 (7)	**18.4** (-5.0 to 41.8; 0%)
	**Patient behaviors not related to care-seeking#**	%	3 / 3 (3)	**16.0** (9.9 to 22.1; 59%)
	**Health worker practice outcomes**	%	9 / 9 (19)	**37.5** (16.7 to 58.3; 96%)
		Cont.	4 / 4 (6)	**4.3** (1.3 to 7.3; 0%)
	**Facilitators (e.g., % of HCPs with job description)**	%	1 / 1 (1)	**non-evaluable****
**QIC: improvement + training** (HF-based HCPs; median follow-up 8.9 months)	**Patient health outcomes**	Cont.	2 / 2 (7)	**73.5** (33.0 to 114.0; 43%)
	**Patient behaviors not related to care-seeking#**	%	2 / 2 (2)	**88.0** (70.3 to 105.6; 94%)
	**Health worker practice outcomes**	%	4 / 4 (10)	**68.1** (53.8 to 82.5; 96%)
		Cont.	1 / 1 (2)	**56.2** (11.7 to 101.2; 0%)
**QIC: improvement + strengthening infrastructure + regulation and governance** (HF-based HCPs; median follow-up 11.0 months)	**Patient behaviors related to care-seeking**	%	2 / 2 (4)	**10.3** (2.9 to 23.5; 99%)
	**Patient behaviors not related to care-seeking#**	%	2 / 2 (4)	**-3.2** (-9.6 to 3.1; 0%)
**QIC: improvement + training** (focused predominantly on lay/ community health workers; median follow-up 14.4 months)	**Health worker practice outcomes**	%	1 / 1 (2)	**50.5** (41.4 to 59.6; 23%)

QIC: Quality improvement collaborative; **HF-based HCPs**: health facility-based health care providers; **95% CI**: 95% confidence interval

* %: outcome expressed as a percentage, Cont.: outcome expressed as continuous and unbounded.

** Meta-analysis was only be performed if the number of effect sizes was > 1 and their standard errors were available.

E.g. patient adherence to treatment regimen.

List of excluded studies

	Citation	Title	Reason for exclusion
1	Ajuzieogu OV, Amucheazi AO. Anesthesia and Analgesia. 2016;123(3):239. doi: 10.1213/01.ane.0000492584.09187.35.	Multidisciplinary collaboration to improve decision-to-incision interval in emergency caesarean sections at a rural community hospital	Ineligible study design
2	Arrieta J, Orrego C, Macchiavello D, Mora N, Delgado P, Giuffre C, García Elorrio E, Rodriguez V. "Goodbye Bacteremia": A Multi-Country Quality Improvement Collaborative Project to Reduce the Incidence of CLABSI in Latin American ICUs. Unpublished manuscript.	"Goodbye Bacteremia": A Multi-Country Quality Improvement Collaborative Project to Reduce the Incidence of CLABSI in Latin American ICUs	Ineligible study design
3	Backman DR, Kohatsu ND, Yu Z, Abbott RE, Kizer KW. Preventing chronic disease. 2017;14:E61. doi: 10.5888/pcd14.160587. PubMed PMID: 28749775.	Implementing a quality improvement collaborative to improve hypertension control and advance million hearts among low-income Californians, 2014-2015	Ineligible setting
4	Balachandran R, Kappanayil M, Sen AC, Sudhakar A, Nair SG, Sunil GS, et al. Annals of Cardiac Anaesthesia. 2015;18(1):52-7. doi: 10.4103/0971-9784.148322. PubMed PMID: 109692187.	Impact of the International Quality Improvement Collaborative on outcomes after congenital heart surgery: a single center experience in a developing economy	Ineligible intervention
5	Barker P, Barron P, Bhardwaj S, Pillay Y. AIDS 2015, 29 (Suppl 2):S137–S143.	The role of quality improvement in achieving effective large-scale prevention of mother-to-child transmission of HIV in South Africa	Ineligible study design
6	Barker PM, Reid A, Schall MW. Implementation Science 2016;11:12.	A framework for scaling up health interventions: lessons from large-scale improvement initiatives in Africa	Ineligible outcomes
7	Bazos DA, LaFave LR, Suresh G, Shannon KC, Nuwaha F, Splaine ME. Implementation science. 2015;10():30. 10.1186/s13012-015-0215-3.	The gas cylinder, the motorcycle and the village health team member: a proof-of-concept study for the use of the Microsystems Quality Improvement Approach to strengthen the routine immunization system in Uganda	Ineligible study design
8	Beutel BG, Jenkins LS. South African Family Practice. 2015;57(2).	Preventing venous thromboembolism at a district hospital: a quality improvement study	Ineligible setting
9	Bookman L, Engmann C, Srofenyoh E, Enweronu-Laryea C, Owen M, Randolph G, Price W, Barker P. Resuscitation. 2010 Sep;81(9):1180-2. Epub 2010 Jul 4.	Educational impact of a hospital-based neonatal resuscitation program in Ghana	Ineligible study design
10	Broughton E, Hermida J, Hill K, Sloan N, Chavez M, Gonzalez D, Freire JM, Gudino X. Front Public Health. 2016 Nov 21;4:247.	Evaluation of an Intervention to Improve Essential Obstetric and Newborn Care Access and Quality in Cotopaxi, Ecuador	Ineligible comparison

	Citation	Title	Reason for exclusion
11	Broughton E, Namajji C, Vaid S, Karamagi-Nkolo E, Byabagambi J. A comparative evaluation and cost-effectiveness analysis of collaborative improvement for maternal and newborn care services in Uganda. Research and Evaluation Report. USAID Health Care Improvement Project. Bethesda (MD): University Research Co., LLC (URC); 2014 Sept.	A comparative evaluation and cost-effectiveness analysis of collaborative improvement for maternal and newborn care services in Uganda. Research and Evaluation Report.	Ineligible comparison
12	Canavan ME, Brault MA, Tatek D, Burssa D, Teshome A, Linnander E, et al. Bulletin of the World Health Organization. 2017;95(6):473-7. doi: 10.2471/BLT.16.178806.	Maternal and neonatal services in Ethiopia: measuring and improving quality	Ineligible study design
13	Cass H. Archives of disease in childhood. 2016;101(5):414-6. doi: 10.1136/archdischild-2015-309007. PubMed PMID: 26729749.	Reducing paediatric medication error through quality improvement networks; where evidence meets pragmatism	Ineligible study design
14	Cassidy J, Ong T, Hill L, Gibson RL. Pediatric Pulmonology. 2015;50:445-6. doi: 10.1002/ppul.23297.	Standardized nursing tool for telephone assessment of pulmonary exacerbation: a quality improvement project	Ineligible setting
15	Chatterjee S, Leese M, Koschorke M, McCrone P, Naik S, John S, Dabholkar H, Goldsmith K, Balaji M, Varghese M, Thara R, Patel V, Thornicroft G; COmmunity care for People with Schizophrenia in India (COPSI) group. Trials. 2011 Jan 13;12:12. doi: 10.1186/1745-6215-12-12. PubMed PMID: 21226970; PubMed Central PMCID: PMC3033834.	Collaborative community based care for people and their families living with schizophrenia in India: protocol for a randomised controlled trial	Ineligible intervention
16	Chatterjee S, Naik S, John S, Dabholkar H, Balaji M, Koschorke M, Varghese M, Thara R, Weiss HA, Williams P, McCrone P, Patel V, Thornicroft G. Lancet. 2014 Apr19;383(9926):1385-94.	Effectiveness of a community-based intervention for people with schizophrenia and their caregivers in India (COPSI): A randomised controlled trial	Ineligible intervention
17	Chirowodza A, Williams D, Diergaardt C, Adetokunboh O, Gede S, Gobodo N, Makeleni N, Tuswa N, Eckard M, O'Rie T, Shingwenyana N, Green B, Oluwatimilehin I. Journal of the International AIDS Society 2016;19():134. 10.7448/IAS.19.6.21264. Abstract TUPDE0102 (page 134 in book of Abstracts from the 21st International AIDS Conference 18–22 July 2016, Durban, South Africa).	The effectiveness of a quality improvement collaborative to accelerate elimination of mother to child transmission (eMTCT): key outcomes and determinants from a demonstration phase collaborative implemented in South Africa, 2012-2015	Ineligible study design

	Citation	Title	Reason for exclusion
18	Chung B, Ngo VK, Ong MK, Pulido E, Jones F, Gilmore J, et al. Psychiatric Services. 2015;66(8):831-9. doi: 10.1176/appi.ps.201400099. PubMed PMID: 109610278.	Participation in training for depression care quality improvement: a randomized trial of community engagement or technical support	Ineligible setting
19	Ciofi degli Atti M, Alegiani SS, Raschetti R, Arace P, Giusti A, Spiazzi R, et al. European Journal of Clinical Pharmacology. 2017;73(9):1141-7. doi: 10.1007/s00228-017-2270-y.	A collaborative intervention to improve surgical antibiotic prophylaxis in children: results from a prospective multicenter study	Ineligible setting
20	Coly A, Sangare K, Broughton E, Djibrina S, Saley Z, Sylla A, Boucar M, Dicko IM, Salem H. 2014. An Evaluation and Cost-effectiveness Analysis of a Collaborative Improvement Intervention for Pre-eclampsia/eclampsia Care in Mali. Research and Evaluation Report. Published by the USAID Health Care Improvement Project. Bethesda MD: University Research Co., LLC (URC).	An Evaluation and Cost-effectiveness Analysis of a Collaborative Improvement Intervention for Pre-eclampsia/Eclampsia Care in Mali. Research and Evaluation Report.	Ineligible study design
21	Dougherty G, Clarke K, Fayorsey R, Kamonga M, Kimambo S, Lutkam D, Madevu-Matson C, Mtiro H, Msuka S, Mugisha V, Panya M, Ramadhani A, Sipemba J, Urasa P, Rabkin M. Journal of the International AIDS Society 2016;19():62-63. Abstract WEAE0106LB (pages 62-63 in book of Abstracts from the 21st International AIDS Conference 18–22 July 2016, Durban, South Africa).	Reaching the first 90: improving coverage of inpatient paediatric provider-initiated HIV testing and counselling using a quality improvement collaborative strategy at 24 health facilities in Tanzania	Ineligible study design
22	Dougherty G, Panya M, Madevu-Matson C, Anyalechi GE, Clarke K, Fayorsey R, et al. J Assoc Nurses AIDS Care. 2019 Feb 18. DOI: 10.1097/JNC.0000000000000066. PMID: 30817370.	Reaching the First 90: improving inpatient pediatric provider-initiated HIV testing and counseling using a quality improvement collaborative strategy in Tanzania	Ineligible outcomes
23	Ducatman AM, Tacker DH, Ducatman BS, Long D, Perrotta PL, Lawther H, et al. Academic pathology. 2017;4:2374289517707506. doi: 10.1177/2374289517707506. PubMed PMID: 28725791.	Quality improvement intervention for reduction of redundant testing	Ineligible setting
24	Evans C, Cho KK, Allen C, Gomez P. International Journal of Gynecology and Obstetrics. 2015;131:E229.	The realities of putting evidence into practice: implementing a low-dose/high-frequency (LDHF) approach to train maternal and newborn care service providers in Ghana, Myanmar, and Uganda	Ineligible intervention

	Citation	Title	Reason for exclusion
25	Fiks AG, Mayne SL, Michel JJ, Miller J, Abraham M, Suh A, et al. Journal of developmental and behavioral pediatrics. 2017;38(8):573-83. doi: 10.1097/dbp.0000000000000490. PubMed PMID: 28816912.	Distance-learning, ADHD quality improvement in primary care: a cluster-randomized trial	Ineligible setting
26	Ford JH, 2nd, Robinson JM, Wise ME. BMC medical education. 2016;16(1):252. doi: 10.1186/s12909-016-0772-4. PubMed PMID: 27681711.	Adaptation of the Grasha Riechman Student Learning Style Survey and Teaching Style Inventory to assess individual teaching and learning styles in a quality improvement collaborative	Ineligible setting
27	Gaies M, MacLaren G. Circulation: Cardiovascular Quality and Outcomes. 2017;10(4). doi: 10.1161/CIRCOUTCOMES.117.003708.	Eliminating postoperative infections on a budget: collaborative quality improvement for congenital heart surgery in low- and middle-income countries	Ineligible setting
28	Gance-Cleveland B, Aldrich H, Schmiege S, Tyler K. International Journal for Quality in Health Care. 2016;28(3):316-23. doi: 10.1093/intqhc/mzw029.	Virtual obesity collaborative with and without decision-support technology	Ineligible setting
29	Garces A, McClure EM, Figueroa L, Pineda S, Hambidge KM, Krebs NF, et al. Reproductive Health. 2016;13:1-8. doi: 10.1186/s12978-016-0178-0. PubMed PMID: 115736486.	A multi-faceted intervention including antenatal corticosteroids to reduce neonatal mortality associated with preterm birth: a case study from the Guatemalan Western Highlands	Ineligible outcomes
30	Hamel DJ, Sankalé JL, Samuels JO, Sarr AD, Chaplin B, Ofuche E, et al. African Journal of Laboratory Medicine. 2015;4(1). doi: 10.4102/ajlm.v4i1.190.	Building laboratory capacity to support HIV care in Nigeria: Harvard/APIN PEPFAR, 2004-2012	Ineligible study design
31	Hsieh FI, Jeng JS, Chern CM, Lee TH, Tang SC, Tsai LK, et al. PLoS ONE. 2016;11(8). doi: 10.1371/journal.pone.0160426.	Quality improvement in acute ischemic stroke care in Taiwan: the breakthrough collaborative in stroke	Ineligible setting
32	Izudi J, Akot A, Kisitu GP, Amuge P, Kekitiinwa A. BioMed Research International. 2016;2016:1-8. doi: 10.1155/2016/5625364. PubMed PMID: 120387076.	Quality improvement interventions for early HIV infant diagnosis in northeastern Uganda	Ineligible study design
33	Izudi J, Epidu C, Katawera A, Kekitiinwa A. BioMed Research International. 2017:1-6. doi: 10.1155/2017/8036535. PubMed PMID: 123303914.	Quality improvement interventions for nutritional assessment among pregnant mothers in northeastern Uganda	Ineligible study design
34	Kc A, Bergstrom A, Chaulagain D, Brunell O, Ewald U, Gurung A, et al. BMJ global health. 2017;2(3):e000497. doi: 10.1136/bmjgh-2017-000497. PubMed PMID: 29071130.	Scaling up quality improvement intervention for perinatal care in Nepal (NePeriQIP); study protocol of a cluster randomised trial	Ineligible intervention

	Citation	Title	Reason for exclusion
35	Kc A, Wrammert J, Clark RB, Ewald U, Vitrakoti R, Chaudhary P, Pun A, Raaijmakers H, Målqvist M. Pediatrics. 2016 Jun;137(6). pii: e20150117. doi:10.1542/peds.2015-0117. PubMed PMID: 27225317.	Reducing perinatal mortality in Nepal using Helping Babies Breathe	Ineligible intervention
36	Kc A, Wrammert J, Nelin V, Clark RB, Ewald U, Peterson S, Målqvist M. BMC Pediatr. 2017 Apr 11;17(1):103. doi: 10.1186/s12887-017-0853-5. PubMed PMID: 28399847; PubMed Central PMCID: PMC5387236.	Evaluation of Helping Babies Breathe Quality Improvement Cycle (HBB-QIC) on retention of neonatal resuscitation skills six months after training in Nepal	Ineligible intervention
37	Khoja S, Scott R, Husyin N, Durrani H, Arif M, Faqiri F, et al. Journal of telemedicine and telecare. 2016;22(8):495-8. doi: 10.1177/1357633x16674631. PubMed PMID: 27799454.	Impact of simple conventional and telehealth solutions on improving mental health in Afghanistan	Ineligible study design
38	Lambeth TM, Rojas MA, Holmes AP, Dail RB. Advances in Neonatal Care. 2016;16(4):264-72. doi: 10.1097/ANC.0000000000000306. PubMed PMID: 117534870.	First Golden Hour of Life: a quality improvement initiative	Ineligible setting
39	Leader A, Cadet C, Lazala D, Roa W, Arroyo O, Jensen L. Frontiers in public health. 2017;5:61. doi: 10.3389/fpubh.2017.00061. PubMed PMID: 28409149.	Collaborative implementation strategy for newborn resuscitation and essential care training in the Dominican Republic	Ineligible study design
40	Leonard W, Uwamahoro D, Uwacu T, Ishimwe A, Mutaganzwa A, Chang E, et al. Journal of the International AIDS Society. 2016;19:135. doi: 10.7448/IAS.19.6.21264.	Effects of continuous quality improvement as a tool for inspiration amongst health care workers and HIV+mothers on rates of HIV and malnutrition amongst HIV-exposed infants in rural Rwanda	Ineligible study design
41	Lumala A, Sekweyama P, Abaasa A, Lwanga H, Byaruhanga R. BMC Pregnancy Childbirth. 2017 Jan 13;17(1):29. doi: 10.1186/s12884-016-1219-y. PubMed PMID: 28086822; PubMed Central PMCID: PMC5237263.	Assessment of quality of care among in-patients with postpartum haemorrhage and severe pre-eclampsia at St. Francis Hospital Nsambya: a criteria-based audit	Ineligible intervention
42	Lyndon A, Cape V. MCN American journal of maternal child nursing. 2016;41(6):E24-e25. doi: 10.1097/nmc.0000000000000291. PubMed PMID: 27759613.	Maternal Hemorrhage Quality Improvement Collaborative Lessons	Ineligible setting
43	Martin C, Naidoo NP, Venter WD, Jaffer A, Barker PM. S Afr Med J. 2014 May 12;104(6):428-30. doi: 10.7196/samj.7507.	Setting ART initiation targets in response to changing guidelines: the importance of addressing both steady-state and backlog	Ineligible outcomes

	Citation	Title	Reason for exclusion
44	Mate KS, Ngidi WH, Reddy J, Mphatswe W, Rollins N, Barker P. BMJ Qual Saf. 2012 Nov 30.	A case report of evaluating a large scale health systems improvement project in an uncontrolled setting: a quality improvement initiative in Kwazu-Natal, South Africa	Ineligible outcomes
45	Mate KS, Ngubane G, Barker PM. Int J Qual Health Care. 2013 Sep;25(4):373-80.	A quality improvement model for the rapid scale-up of a program to prevent mother-to-child HIV transmission in South Africa	Ineligible outcomes
46	Morris C, Alexander I. BMJ quality improvement reports. 2016;5(1). doi: 10.1136/bmjquality.u212664.w5045. PubMed PMID: 27752316.	Developing quality improvement capacity and capability across the Children in Fife partnership	Ineligible setting
47	Naidoo K, Sitharam L. Southern African Journal of Epidemiology and Infection. 2015;30(3):147.	Collaborative approach to decreasing the central line-associated blood stream infection rate (CLABSI) at a private hospital in KZN	Ineligible study design
48	Patel V, Weiss HA, Chowdhary N, Naik S, Pednekar S, Chatterjee S, Bhat B, Araya R, King M, Simon G, Verdeli H, Kirkwood BR. Br J Psychiatry. 2011 Dec;199(6):459-66.	Lay health worker led intervention for depressive and anxiety disorders in India: impact on clinical and disability outcomes over 12 months	Ineligible intervention
49	Patel V, Weiss HA, Chowdhary N, Naik S, Pednekar S, Chatterjee S, De Silva MJ, Bhat B, Araya R, King M, Simon G, Verdeli H, Kirkwood BR. Lancet. 2010 Dec 18;376(9758):2086-95. doi: 10.1016/S0140-6736(10)61508-5. Epub 2010 Dec 13. PubMed PMID: 21159375; PubMed Central PMCID: PMC4964905.	Effectiveness of an intervention led by lay health counsellors for depressive and anxiety disorders in primary care in Goa, India (MANAS): a cluster randomised controlled trial	Ineligible intervention
50	Patel VH, Kirkwood BR, Pednekar S, Araya R, King M, Chisholm D, Simon G, Weiss H. Trials. 2008 Jan 25;9:4. doi:10.1186/1745-6215-9-4. PubMed PMID: 18221516; PubMed Central PMCID: PMC2265673.	Improving the outcomes of primary care attenders with common mental disorders in developing countries: a cluster randomized controlled trial of a collaborative stepped care intervention in Goa, India	Ineligible intervention
51	Rakhmanova NA, Snidevich U, Semenenko I. International Journal of Infectious Diseases. 2016;45:162. doi: 10.1016/j.ijid.2016.02.387.	Improving HIV service delivery in detention centers and ART facility in Odessa, Ukraine	Ineligible study design
52	Rankin K, Gavin L, Moran J, Kroelinger C, Vladutiu C, Goodman D, et al. Maternal & Child Health Journal. 2016;20(11):2239-46. doi: 10.1007/s10995-016-2105-y. PubMed PMID: 119356379.	Importance of performance measurement and MCH epidemiology leadership to quality improvement initiatives at the national, state and local levels	Ineligible study design
53	Rivaz M, Momennasab M, Shokrollahi P. J Adv Med Educ Prof. 2015 Oct;3(4):178-82. PubMed PMID: 26457315; PubMed Central PMCID:PMC4596384.	Effect of collaborative testing on learning and retention of course content in nursing students	Ineligible intervention

	Citation	Title	Reason for exclusion
54	Rule ARL, Maina E, Cheruiyot D, Mueri P, Simmons JM, Kamath-Rayne BD. Acta Paediatrica. 2017;106(10):1666-73. doi: 10.1111/apa.13940. PubMed PMID: 125011639. .	Using quality improvement to decrease birth asphyxia rates after 'Helping Babies Breathe' training in Kenya	Ineligible study design
55	Salins N, Johnson J, Macaden S. Indian journal of palliative care. 2017;23(1):3-12. doi: 10.4103/0973-1075.197952. PubMed PMID: 28216856.	Feasibility and acceptability of implementing the Integrated Care Plan for the Dying in the Indian setting: survey of perspectives of Indian palliative care providers	Ineligible study design
56	Savino, T. Hospital peer review. 2016;41(1):7-8.	Hospital's sepsis QI program lead to drop in mortality rate	Ineligible setting
57	Sen AC, Morrow DF, Balachandran R, Du X, Gauvreau K, Jagannath BR, et al. Circulation: Cardiovascular Quality and Outcomes. 2017;10(4). doi: 10.1161/CIRCOUTCOMES.116.002935.	Postoperative infection in developing world congenital heart surgery programs: data from the International Quality Improvement Collaborative	Ineligible intervention
58	Sifrim ZK, Barker PM, Mate KS. BMJ Qual Saf. 2012 May;21(5):423-31. doi: 10.1136/bmjqs-2011-000445. Epub 2012 Mar 23. Erratum in: BMJ Qual Saf. 2012 Nov;21(11):972. PubMed PMID: 22447823.	What gets published: the characteristics of quality improvement research articles from low- and middle-income countries	Ineligible study design
59	Uwimana J, Zarowsky C, Hausler H, Jackson D. BMC Health Serv Res. 2012 Aug 2;12:233.	Engagement of non-government organisations and community care workers in collaborative TB/HIV activities including prevention of mother to child transmission in South Africa: opportunities and challenges	Ineligible intervention
60	Weaver MR, Burnett SM, Crozier I, Kinoti SN, Kirunda I, Mbonye MK, Naikoba S, Ronald A, Rubashembusya T, Zawedde S, Willis KS. PLoS One 2014 Aug 18;9(8):e103017.	Improving facility performance in infectious disease care in Uganda: a mixed design study with pre/post and cluster randomized trial components	Ineligible comparison
61	Wilkinson L, Harley B, Sharp J, Solomon S, Jacobs S, Cragg C, Kriel E, Peton N, Jennings K, Grimsrud A. Trop Med Int Health. 2016 Jun;21(6):743-9. doi: 10.1111/tmi.12699.	Expansion of the Adherence Club model for stable antiretroviral therapy patients in the Cape Metro, South Africa 2011-2015	Ineligible study design
62	Youngleson MS, Nkurunziza P, Jennings K, Arendse J, Mate KS, Barker P. PLoS ONE. 2010;5(11):e13891.	Improving a mother to child HIV transmission programme through health system redesign: quality improvement, protocol adjustment and resource addition	Ineligible study design
